# Targeting Egfr‐Mediated Cell Proliferation and Lipid Metabolism Separation Effectively Accelerate Liver Regeneration

**DOI:** 10.1111/cpr.70214

**Published:** 2026-04-22

**Authors:** Yuelei Hu, Shifei Song, Ruilin Wang, Ni An, Jinmei Diao, Yuguo Chen, Juan Liu, Guoyue Lv

**Affiliations:** ^1^ Department of Hepatobiliary and Pancreatic Surgery, General Surgery Center First Hospital of Jilin University Changchun Jilin China; ^2^ Department of Cadre's Wards Ultrasound Diagnostics. Ultrasound Diagnostic Center The First Hospital of Jilin University Changchun Jilin China; ^3^ Hepato‐Pancreato‐Biliary Center, Beijing Tsinghua Changgung Hospital, School of Clinical Medicine, Tsinghua Medicine Tsinghua University Beijing China; ^4^ School of Future Medicine Beijing University of Chinese Medicine Beijing China; ^5^ China‐Singapore Belt and Road Joint Laboratory on Liver Disease Research Changchun China

**Keywords:** Acsl1, lipid metabolism, liver regeneration, metabolic reprogramming, Ppara

## Abstract

Hepatocyte proliferation restores liver mass after partial hepatectomy (PHx), but the metabolic cost of this process remains unclear. Single‐nucleus transcriptomics of mouse liver 48 h after 70% PHx revealed that EGFR–FOXM1 signalling drives mitotic entry while simultaneously suppressing PPARα–ACSL1‐mediated lipid catabolism. Consequently, triglycerides and free fatty acids accumulate in regenerating tissue. Activating PPARα with the agonist Wy‐14643 released this metabolic brake, accelerated hepatocyte proliferation via HIF1α‐FOXM1, and improved post‐PHx recovery. These data identify lipid‐metabolic reprogramming as an EGFR‐dependent collateral effect that can be pharmacologically reversed to enhance liver regeneration in surgical patients, offering a readily translatable strategy to reduce post‐operative liver failure and shorten hospital stay after major hepatectomy.

## Graphical abstract

AREG coordinates cell proliferation and lipid metabolism reprogramming during liver regeneration and targeting lipid metabolism can promote liver regeneration.

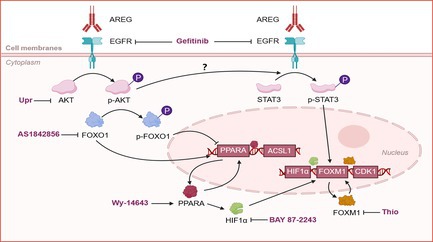

## 1 Introduction

Partial hepatectomy (PHx) is a primary therapeutic intervention for liver tumours. Following PHx, the remnant liver undergoes rapid and extensive regeneration to restore its original mass and function, ensuring the body's metabolic and synthetic homeostasis [1]. The mechanisms governing liver regeneration (LR) are highly complex, involving not only hepatocytes but also non‐parenchymal cells (NPCs) and even inter‐organ crosstalk [2, 3, 4]. Critical transcription factors (TFs) such as Yap, Foxm1 and Stat3 are activated within hepatocytes to drive the expression of proliferation‐associated genes [3, 5]. Concurrently, NPCs—including endothelial cells, hepatic stellate cells and kupffer cells—respond to mechanical and metabolic stimuli by secreting pro‐proliferative ligands, such as those in the Egfr and Wnt families, which are pivotal for initiating and sustaining the regenerative response [2, 6, 7].

Significant metabolic reprogramming occurs during LR [8, 9]. Transient regenerative‐associated steatosis (TRAS), a prominent metabolic feature, has been historically attributed primarily to the influx of free fatty acids (FFAs) derived from lipolysis in peripheral adipose tissue, which are subsequently taken up by hepatocytes. These lipid droplets (LDs) and accumulated fatty acids are utilised by proliferating hepatocytes as an energy source via oxidative metabolism [10, 11]. Additionally, as early as 6 h after PHx, hepatic accumulation of acetyl‐CoA can be observed. This rise in acetyl‐CoA levels enhances histone H3K27 acetylation, which in turn epigenetically regulates the expression of genes critical to the regenerative process [12]. These findings highlight the intricate role of metabolic reprogramming in orchestrating LR.

In healthy adults, hepatocytes are typically quiescent. Injury‐induced loss of hepatocytes triggers the remaining cells to re‐enter the cell cycle in a tightly controlled manner, which is fundamental for tissue repair. However, under chronic pathogenic conditions, this controlled proliferation can become dysregulated, leading to malignant transformation and hepatocellular carcinoma (HCC). From a broader perspective, both HCC cells and regenerating hepatocytes can be categorised as ‘proliferative hepatocytes’. Such proliferating hepatocytes undergo significant metabolic reprogramming alongside active cell division, features that have been well‐documented in both LR and HCC research [8, 13]. Multi‐omics studies in HCC have revealed significant suppression of lipid metabolism and PPAR signalling pathway in tumour tissues compared to adjacent non‐tumour liver tissues [14]. Similarly, single‐cell transcriptomic analyses of regenerating mouse livers after 70% PHx demonstrated that lipid metabolic processes are markedly inhibited during the early phase of regeneration, followed by gradual recovery as regeneration proceeds [9]. These observations suggest that metabolic reprogramming is a universal characteristic of proliferating hepatocytes.

Although multi‐omics technologies and genetically engineered animal models have substantially advanced our understanding of LR, there are still no clinically available therapies that effectively enhance regenerative capacity. Lipid metabolic reprogramming is now recognised as a deterministic event during LR; however, whether targeting lipid metabolism can improve regenerative outcomes—and through what mechanisms—remains unclear. Therefore, using a mouse model of 70% PHx, we aim to integrate single‐nucleus transcriptomics and bulk RNA sequencing to systematically investigate the dynamics of hepatocyte proliferation and metabolic reprogramming during LR. This study seeks to identify potential metabolic targets that may enhance liver repair and to elucidate the mechanisms by which modulation of lipid metabolism promotes regenerative processes.

## 2 Results

### 2.1 Egfr‐Mediated Proliferation and Stemness in Proliferative Hepatocyte Subpopulations

**FIGURE 1:**
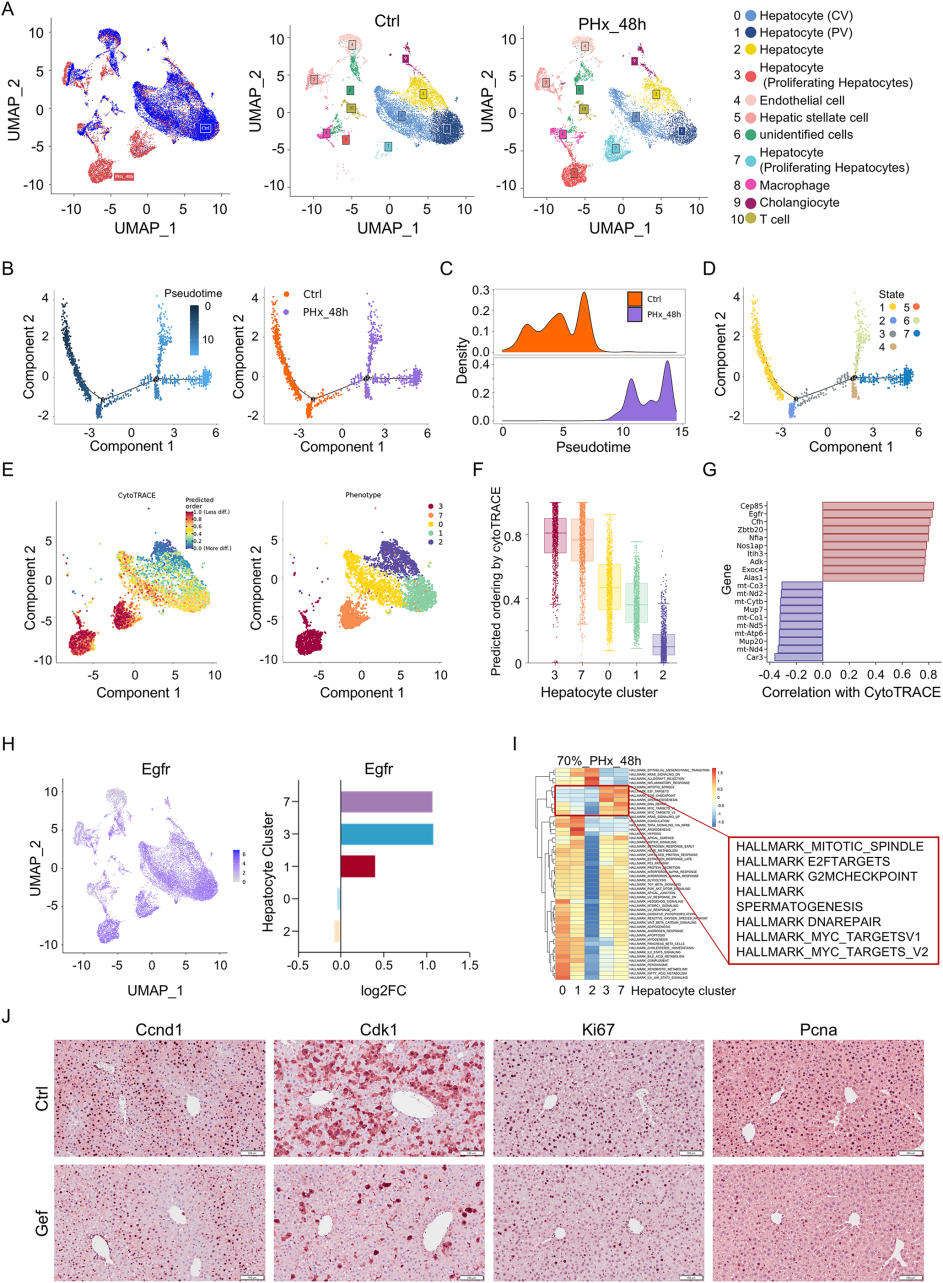
Egfr mediates hepatocyte proliferation during LR. (A) UMAP plots showing the distribution and density of specific cell clusters at 0 and 48 h, visualising spatial changes in the transcriptional landscape. (B, C) Pseudotime analysis reveals the differences in hepatocyte states between the Ctrl and PHx‐48 h groups. (D) Pseudotime plot indicating cellular trajectories of hepatocytes from all samples. Single‐cell trajectories were constructed and pseudotime values were calculated using Monocle 2. Tajectories are coloured by pseudotime. (E) CytoTRACE analysis was performed to assess the stemness of the five hepatocyte subpopulations. UMAP projection shows the mapping of CytoTRACE‐predicted stemness values. (F) Distribution of CytoTRACE scores across the five hepatocyte clusters. (G) Top 10 genes associated with higher differentiation states (blue) and lower differentiation states (pink; higher stemness). (H) Expression and distribution patterns of Egfr across the five hepatocyte subpopulations. (I) GSVA enrichment score heatmap indicates that Clusters 3 and 7 are primarily associated with biological processes such as mitosis and cell cycle. (J) Gefitinib, an Egfr inhibitor, effectively inhibits the LR process following 70% PHx (*n* = 3).

To investigate the alterations in proliferative and metabolic patterns of hepatocytes following 70% PHx in mice, we established a 70% PHx model. A gradual increase in the liver‐to‐body weight ratio indicated successful surgery and initiation of LR (Figure S1A). We then performed single‐nucleus RNA sequencing (snRNA‐seq) on right lobe liver tissues from both control (Ctrl) and 48 h post‐hepatectomy (PHx_48h) groups. Uniform Manifold Approximation and Projection (UMAP) analysis identified ten distinct cell clusters (Figure S1B). Among these, five clusters represented hepatocyte subpopulations, with two clusters (Clusters 3 and 7) exhibiting pronounced proliferative characteristics, as evidenced by elevated expression of genes such as Mki67, Fxom1, Cdk1 and Cdc20 (Figure S1C). Comparative analysis revealed that Clusters 3 and 7 were predominantly present in the PHx_48h group. In contrast, the proportions of Clusters 0, 1 and 2 were significantly reduced at 48 h after surgery, with the most notable decrease observed in Cluster 1, which represents periportal hepatocytes (Figure S1D). The shift in cellular composition between Ctrl and PHx_48h was clearly visualised in the UMAP embedding (Figure 1A). Further supporting their proliferative status, Clusters 3 and 7 showed high expression of mitotic markers including Mki67, Kif20a, Cdc20, Ccnb1 and Plk1 (Figure S1E). Immunohistochemistry (IHC) results indicated spatially heterogeneous proliferation: staining for CyclinD1 revealed that periportal hepatocytes were the first to enter the cell cycle (Figure S1F), consistent with the pronounced reduction in the proportion of this subpopulation.

To delineate the transcriptional transition of hepatocytes from quiescence to proliferation, we performed pseudotime analysis on hepatocytes using Monocle2 to compare cells from both Ctrl and PHx_48h groups. The results indicated that hepatocytes from the Ctrl group occupied an earlier pseudotemporal state compared to those from the PHx_48h group (Figure 1B,C). The pseudotime trajectory categorised all hepatocytes into seven distinct states: States 1–3 predominantly consisted of Ctrl hepatocytes, whereas States 4–7 were largely comprised of PHx_48h hepatocytes. Further analysis of the distribution of clusters 0, 1, 2, 3 and 7 along the pseudotime trajectory revealed that Clusters 3 and 7 were positioned at later pseudotemporal stages (Figures 1D and S2). Given that proliferative capacity is a hallmark of stem cells, we asked whether hepatocytes re‐entering the cell cycle also acquire stem‐like properties. To assess this, we applied CytoTRACE to evaluate the stemness features of the five hepatocyte subpopulations. The analysis showed that Clusters 3 and 7 exhibited significantly higher stemness scores compared to clusters 0, 1 and 2 (Figure 1E,F). To investigate the underlying factors associated with enhanced stemness in Clusters 3 and 7, we identified several genes positively correlated with stemness, including Cep85, Egfr, Cfh, Zbtb20, Nfia, Nos1ap, Itih3, Adk, Exoc4 and Alas1 (Figure 1G). Among these, Egfr—a key cell surface receptor in hepatocytes—plays a critical role in mediating microenvironmental signals and promoting hepatocyte proliferation. We first examined the expression of Egfr across hepatocyte subpopulations and found it to be highly expressed in proliferating clusters (Figure 1H). GSVA enrichment analysis of hallmark gene sets revealed that genes upregulated in Clusters 3 and 7 were significantly enriched in processes such as MITOTIC_SPINDLE, E2F_TARGETS, G2M_CHECKPOINT, SPERMATOGENESIS, DNA_REPAIR, MYC_TARGETS_V1 and MYC_TARGETS_V2 (Figure 1I), further supporting a role for Egfr in driving proliferation. Functional validation using the Egfr‐specific inhibitor gefitinib effectively suppressed LR following 70% PHx in mice (Figure 1J). In summary, these findings demonstrate that Egfr plays an essential role in promoting both proliferation and the acquisition of stem‐like properties in hepatocytes during LR.

### 2.2 Egfr‐Foxm1 Signalling Axis Orchestrates the Transcriptional Program Controlling Hepatocyte Proliferation

**FIGURE 2:**
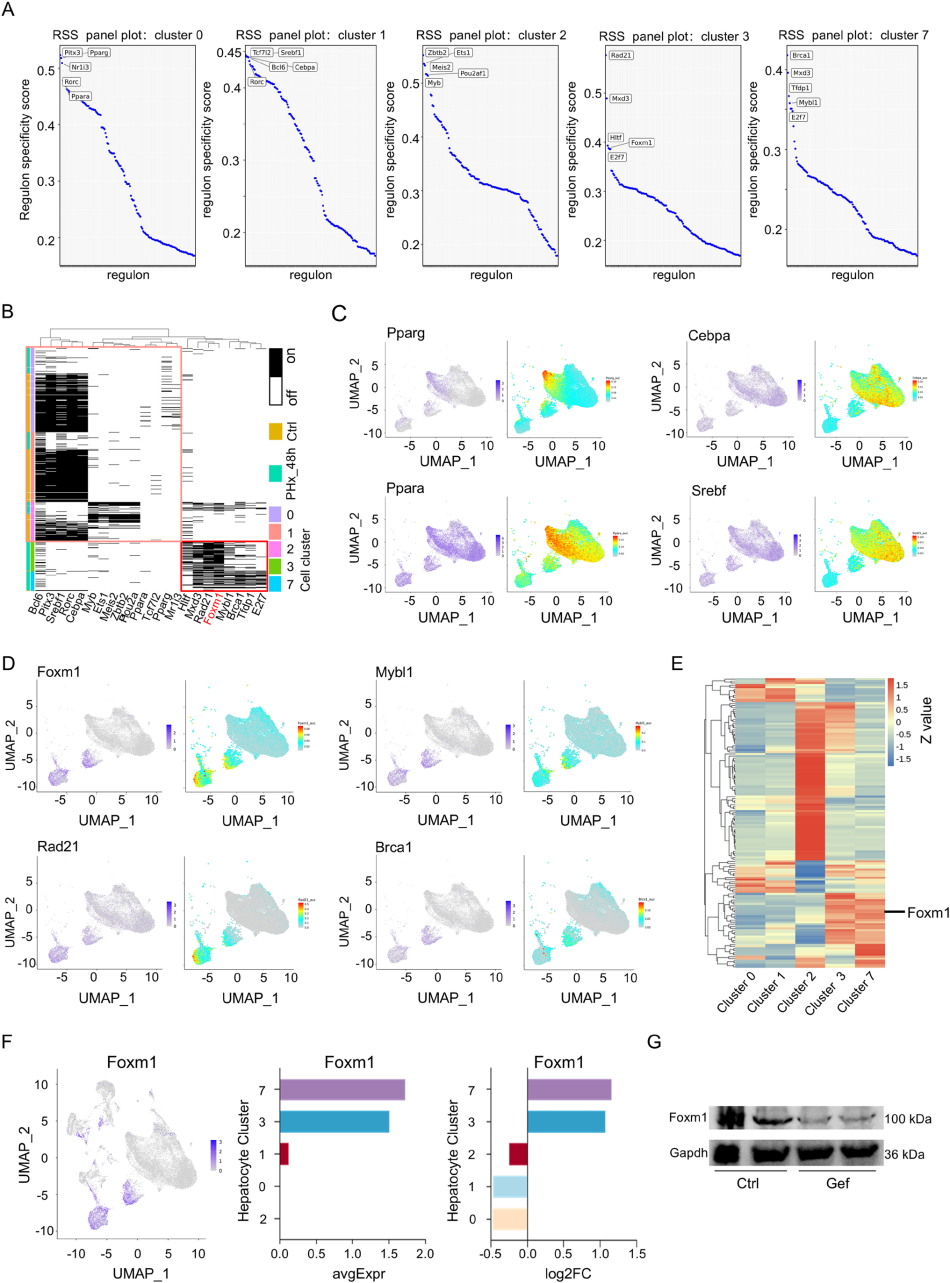
sn‐RNA seq analysis identifies Foxm1 as a core transcription factor downstream of EGFR activation, mediating the transcriptional regulation of proliferating hepatocytes. (A) Top 5 transcription factors ranked by Regulon Specificity Score (RSS) within different hepatocyte subclusters. (B) Heatmap showing the differential activity of the top 5 Regulons across cell clusters, based on RSS calculation. (C) FeaturePlot visualising the expression and AUC values of selected Regulons in non‐proliferating hepatocytes, calculated based on RSS. (D) FeaturePlot visualising the expression and AUC values of selected Regulons in proliferating hepatocytes, calculated based on RSS. (E) Heatmap of *Z*‐scores for Regulon activity across different hepatocyte subpopulations. *Z*‐scores represent the standardised mean AUCell values per cell type using the overall mean and standard deviation. (F) Distribution and differential expression of Foxm1 across various cell subpopulations. (G) Gefitinib (an Egfr inhibitor) suppresses the expression of Foxm1 during the process of LR (*n* = 3).

We further analysed TF activity during LR. SCENIC (single‐cell regulatory network inference and clustering) was employed to infer TF activity across all hepatocyte subpopulations. The results revealed distinct TF activity patterns: the most active TFs in Cluster 0 included Pitx3, Pparg, Nr1i3, Rorc and Ppara; in Cluster 1, Tcf7l2, Srebf1, Bcl6, Cebpa and Rorc were most active; Cluster 2 exhibited high activity of Zbtb2, Ets1, Meis2, Pou2af1 and Myb; Cluster 3 showed prominent activity of Rad21, Mxd3, Hltf, Foxm1 and E2f7; and Cluster 7 was characterised by high activity of Brca1, Mxd3, Tfdp1, Mybl1 and E2f7 (Figure 2A). Notably, TF activity profiles differed markedly among hepatocyte subpopulations. Proliferating hepatocyte clusters (e.g., Clusters 3 and 7) exhibited elevated activity of TFs such as Rad21, Foxm1, Brca1 and Mybl1, whereas non‐proliferating hepatocyte clusters were associated with higher activity of Pparg, Ppara, Srebf1 and Cebpa. Interestingly, certain TFs that were in an ‘ON’ state in Clusters 0, 1 and 3 under Ctrl group switched to an ‘OFF’ state in the PHx_48h group (Figure 2B). TF activities were highly specific in different hepatocyte clusters (Figures 2C,D and S3). *Z*‐scores were used to evaluate the influence of TFs on gene expression. A heatmap of *Z*‐scores for all regulons across hepatocyte subpopulations indicated that the Foxm1‐mediated regulon was among the most active in proliferating hepatocytes (Figure 2E). Consistent with this, Foxm1 expression was highest in the proliferating Clusters 3 and 7 (Figure 2F). Given our previous finding that Egfr is a key regulator of proliferation and stemness maintenance in Clusters 3 and 7, we asked whether Egfr activation regulates Foxm1 expression. Western blot analysis showed that treatment with gefitinib, a specific Egfr inhibitor, significantly suppressed Foxm1 protein levels (Figures 2G and S7A). This suggests that the transcriptional regulatory effects mediated by Foxm1 are partially downstream of EGFR activation. In summary, Foxm1 transcriptional activity is markedly elevated in proliferating hepatocytes, and its regulatory role is partly attributable to biological effects initiated by Egfr signalling.

### 2.3 Foxm1 Orchestrates LR Through Transcriptional Activation of Target Genes

**FIGURE 3:**
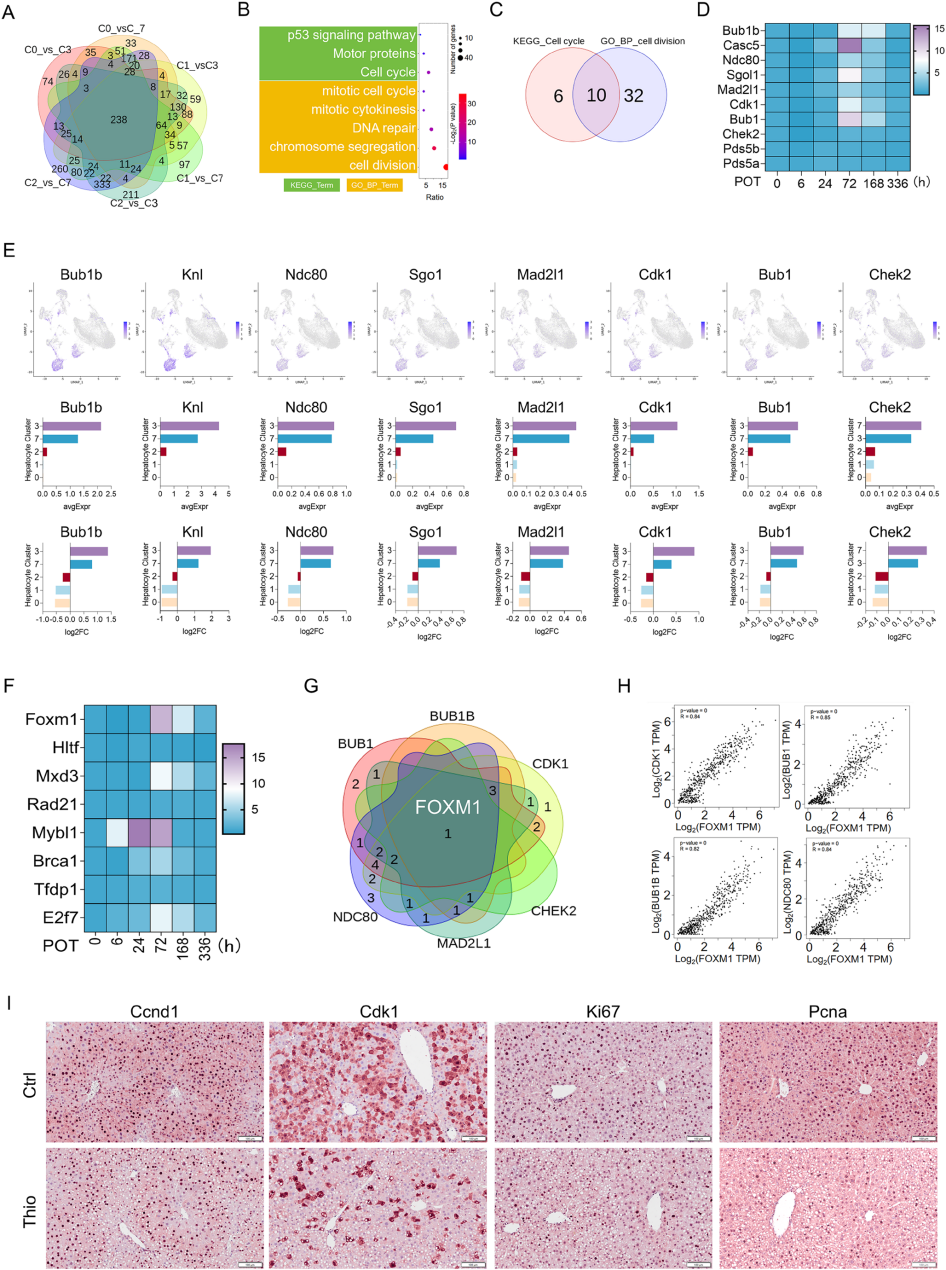
Foxm1 transcriptionally regulates the expression of key genes in proliferating hepatocytes. (A) A total of 238 genes showed higher expression in Clusters 3 and 7 compared to Clusters 0, 1 and 2. (B) KEGG and GO Biological Process (GO_BP) enrichment analysis of the 238 differentially expressed genes. (C) Identification of 10 overlapping genes significantly associated with both the ‘KEGG_CELL_CYCLE’ and ‘GOBP_CELL_DIVISION’ terms. (D) Dynamic expression changes of the 10 key genes during the process of LR. (E) Expression distribution and differences of the key genes across various hepatocyte subpopulations. (F) Expression differences of the active transcription factors in proliferating hepatocytes during LR after 70% PHx. (G) Prediction of transcription factor‐target relationships reveals Foxm1 as a key upstream regulator of the identified target genes. (H) Correlation analysis between Foxm1 and the key target genes in human HCC tissue samples from the GEPIA database. (I) Immunohistochemical results demonstrate that a Foxm1 inhibitor effectively suppresses the LR process (*n* = 3).

To further elucidate the key gene regulatory mechanisms mediating hepatocyte proliferation, we identified differentially expressed genes between proliferating (Clusters 3 and 7) and non‐proliferating (Clusters 0, 1 and 2) hepatocyte subpopulations in the PHx_48h group. A total of 238 genes were consistently up‐regulated in both proliferating clusters (Figure 3A). GO_BP and KEGG enrichment analyses of these genes revealed significant associations with cell cycle and cell division processes (Figure 3B). Among the terms ‘KEGG_cell cycle’ and ‘GO_BP_cell division’, 10 key overlapping genes were identified: Bub1b, Knl1 (Casc5), Ndc80, Sgo1 (Sgol1), Mad2l1, Cdk1, Bub1, Chek2, Pds5b and Pds5a (Figure 3C). Expression dynamics of these genes during mouse LR are displayed in Figure 3D. Furthermore, these genes exhibited elevated and specific expression in Clusters 3 and 7 (Figure 3E). To investigate the regulatory mechanisms controlling these key genes, we analysed bulk transcriptome data from regenerating liver tissues after 70% PHx, focusing on TFs active in Clusters 3 and 7 (as identified in Figure 1). This analysis indicated that Foxm1 and Mybl1 underwent the most pronounced changes (Figure 3F). In addition, prediction using online web site databases suggested that Foxm1 is a common potential regulator of Bub1b, Mad2l1, Ndc80, Cdk1, Bub1 and Chek2 (Figures 3G and S4A). Consistent with this, analysis via the GEPIA platform revealed strong correlations between FOXM1 expression and that of BUB1B, NDC80, CDK1, BUB1, SGO1 (SGOL1), MAD2L1, CHEK2 and KNL1 (CASC5) (Figures 3H and S4B). To functionally validate these findings, mice undergoing 70% PHx were treated with Thio, a Foxm1‐specific inhibitor. The results demonstrated that Thio effectively impaired LR and downregulated the expression of key genes such as Cdk1 (Figure 3I). In summary, these results indicate that ten core genes, including Cdk1, play crucial roles in LR and are transcriptionally regulated by Foxm1. These data underscore the importance of the Egfr–Foxm1 axis in regulating hepatocyte proliferation during LR.

### 2.4 Integrated Transcriptomic Profiling Identifies Metabolic Reprogramming During LR

**FIGURE 4:**
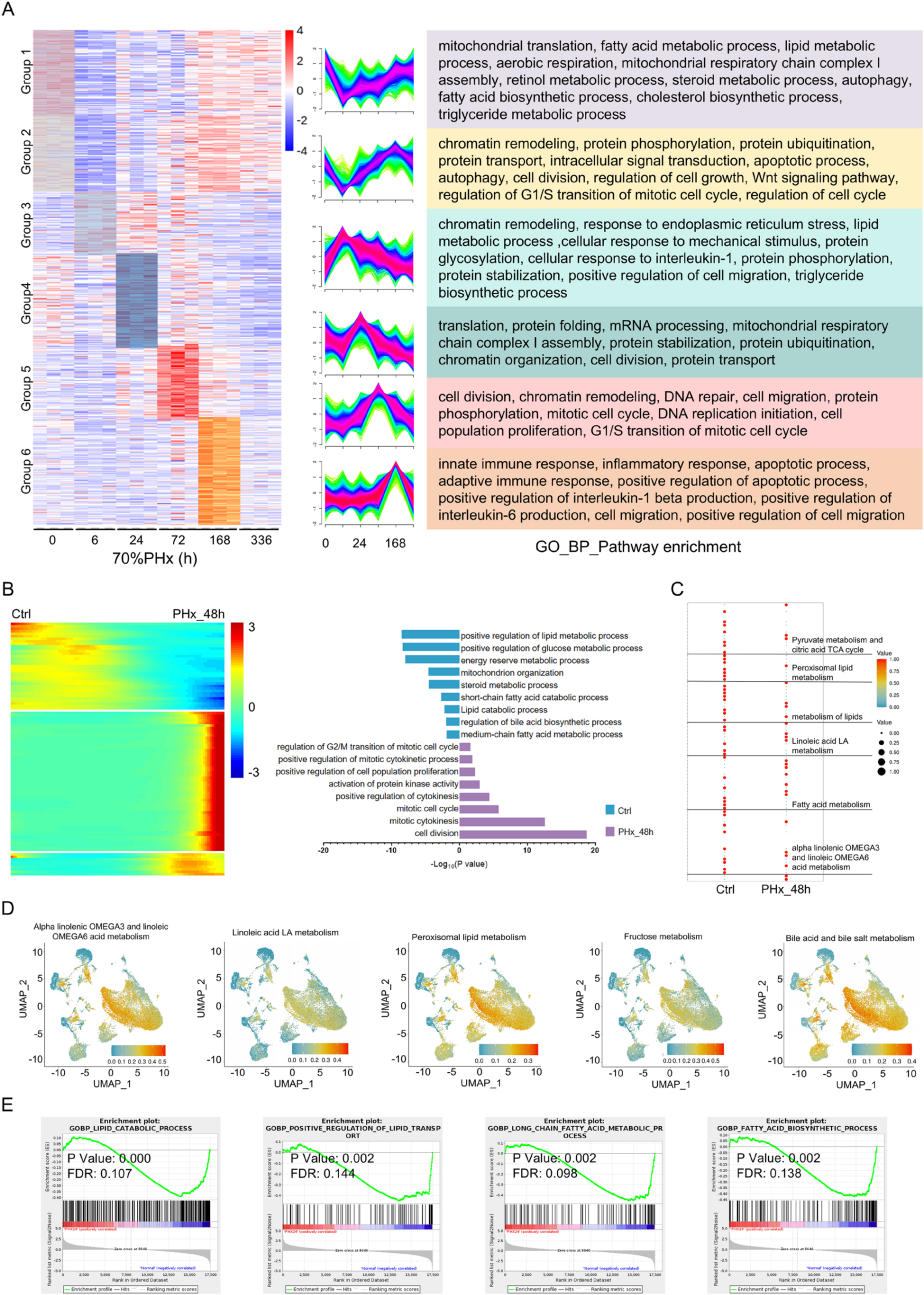
Integrated transcriptomic analysis reveals significant suppression of lipid metabolism in proliferating hepatocytes during LR. (A) Enrichment analysis of preferentially expressed genes and signalling pathways at different time points after 70% PHx in mice. (B) Heat maps representing modules of genes that co‐vary along the pseudotime during Ctrl and PHx_48h phases. The representative up‐regulated and down‐regulated GO terms are described beside the heat maps. (C) scMetabolism analysis demonstrates a reduction in lipid metabolism during LR after 70% PHx. (D) UMAP visualisation shows differences in activity scores of representative metabolic pathways per cell across distinct hepatocyte subpopulations. (E) Gene Set Enrichment Analysis (GSEA) confirms the inhibition of lipid metabolism pathways following 70% PHx, comparing 24 and 0 h time points.

Although multiple studies have indicated that metabolic reprogramming occurs during LR, it remains unclear whether proliferating hepatocytes, non‐proliferating hepatocytes or both undergo metabolic reprogramming, and what specific metabolic adaptations take place in each population. To investigate this, we analysed bulk transcriptome sequencing data from regenerating liver tissues after 70% PHx available in the GEO database. We observed distinct patterns of preferentially expressed genes at different time points during regeneration. Differentially expressed genes were clustered into six groups. Genes in Group 1 were predominantly enriched in mitochondrial translation, fatty acid metabolic process, lipid metabolic process, aerobic respiration, mitochondrial respiratory chain complex I assembly, fatty acid biosynthetic process and triglyceride (TG) metabolic process, indicating marked suppression of lipid metabolism and mitochondrial function in the early regenerative phase. Group 2 was largely associated with cell cycle and division, suggesting that proliferative programmes are also initially repressed. Highly expressed genes at 6 h post‐PHx (Group 3) were enriched in chromatin remodelling, response to endoplasmic reticulum stress, lipid metabolic process, cellular response to mechanical stimulus, cellular response to interleukin‐1, protein phosphorylation, protein stabilisation and TG biosynthetic process, implying that hepatocytes begin to perceive physical (e.g., mechanical) and chemical (e.g., cytokine) signals from the microenvironment. At 24 h (Group 4), highly expressed genes were related to translation, protein folding, mRNA processing, mitochondrial respiratory chain Complex I assembly, protein stabilisation, protein ubiquitination, chromatin organisation, cell division and protein transport, suggesting enhanced protein synthesis, improved mitochondrial respiration and initiation of cell division. At 72 h (Group 5), genes involved in cell division, chromatin remodelling, DNA repair, cell migration, protein phosphorylation, mitotic cell cycle, DNA replication initiation, cell population proliferation and G1/S transition of mitotic cell cycle were upregulated, indicating active cell proliferation (Figure 4A). We next performed pseudotime trajectory‐based co‐expression analysis, which revealed that genes associated with cell proliferation and lipid metabolism were expressed in distinct hepatocyte subpopulations (Figure 4B). Using scMetabolism to evaluate metabolic alterations at single‐cell resolution, we observed significant downregulation in the PHx_48h group of multiple pathways including pyruvate metabolism and citric acid (TCA) cycle, peroxisomal lipid metabolism, metabolism of lipids, linoleic acid (LA) metabolism, fatty acid metabolism and metabolism of alpha‐linolenic (OMEGA3) and linoleic (OMEGA6) acids, based on Reactome gene sets (Figures 4C and S5A). In non‐proliferating hepatocytes, PPARα expression was markedly predominant (Figure S5B). Metabolic activity scoring further confirmed pronounced suppression of these processes specifically in proliferating hepatocytes (Figure 4D). GSEA of bulk transcriptome data comparing 24 and 0 h after PHx also indicated significant inhibition of lipid metabolism‐related pathways (Figures 4E and S5C–I). In summary, these results demonstrate that extensive metabolic reprogramming occurs following 70% PHx, with lipid metabolic reprogramming being a major component. snRNA‐seq analysis further indicates that this metabolic shift occurs primarily in proliferating hepatocytes, which exhibit severe suppression of lipid metabolism.

### 2.5 Metabolic Reprogramming via the AREG‐EGFR‐AKT‐FOXO1‐Mediated Suppression of PPARA‐ACSL1

**FIGURE 5:**
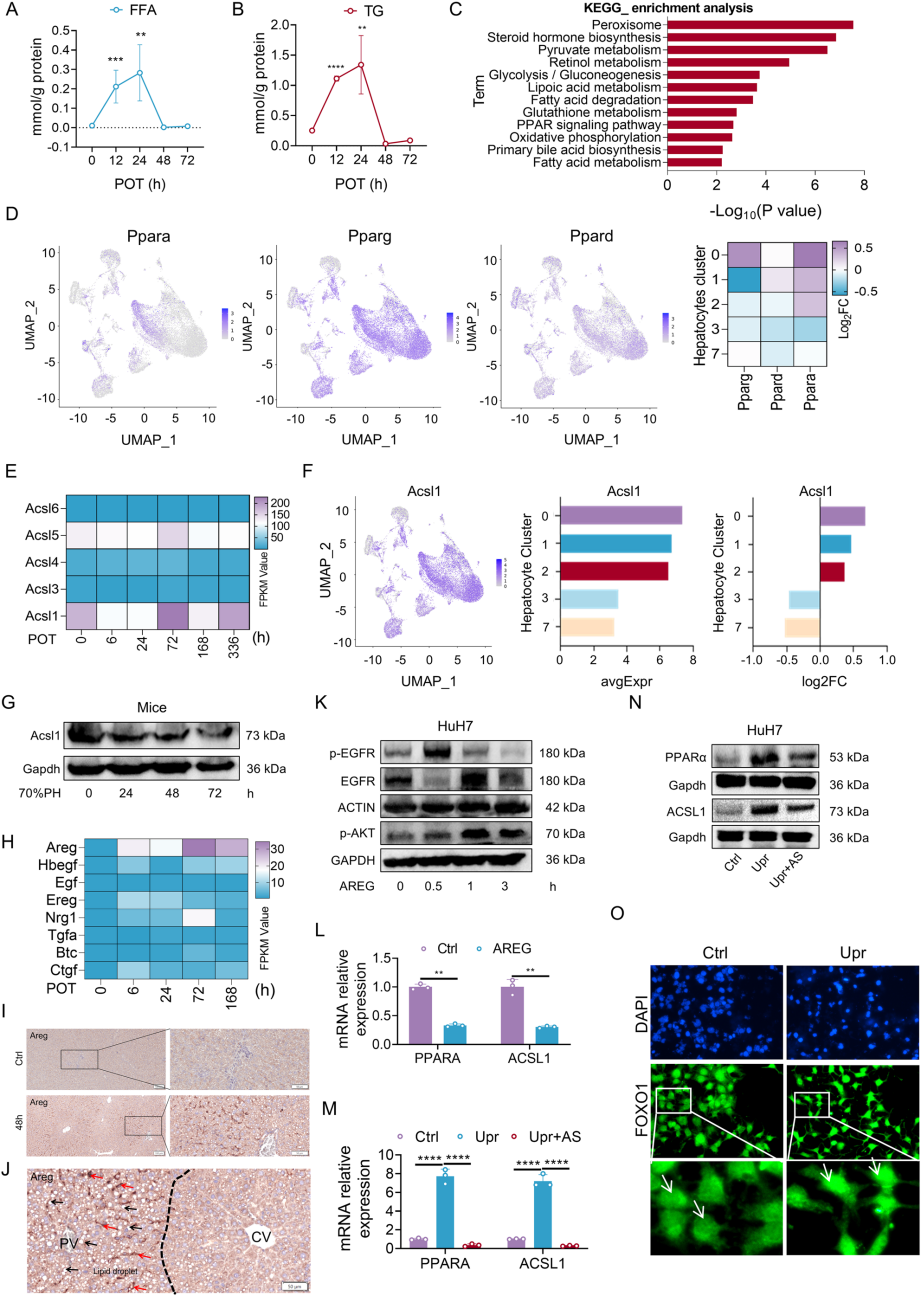
The AREG‐EGFR‐AKT‐FOXO1 axis mediates lipid metabolic reprogramming during LR by suppressing PPARA‐ACSL1 expression. (A, B) Changes in fatty acid and triglyceride levels in residual liver tissue during regeneration after 70% PHx in mice. (C) KEGG enrichment analysis of the Group 1 gene cluster from Figure 4A shows decreased activity in peroxisome, PPAR signalling and lipid metabolism‐related pathways during regeneration. (D) Distribution and differential activity of Ppara, Pparg and Ppard across different cell clusters. (E) Expression changes of Acsl family genes during LR. (F) Expression and distribution of Acsl1 across different cell populations. (G) Western blot analysis shows decreased Acsl1 protein expression during LR. (H) Expression changes of common EGFR ligands during LR. (I) IHC analysis revealed a significant increase in AREG expression post‐surgery, with predominant localisation to hepatic sinusoidal endothelial cells. (J) Within the hepatic lobule, AREG is predominantly expressed in sinusoidal endothelial cells of the PV zone (red arrows), whereas lipid droplet accumulation is more pronounced in PV zone hepatocytes (black arrows). (K) AREG promotes phosphorylation of EGFR and AKT in the human hepatoma cell line Huh7. (L) q‐RT‐PCR results show that AREG suppresses the expression of Ppara and Acsl1 in Huh7 cells. (M, N) q‐RT‐PCR and Western blot analysis demonstrates that an AKT inhibitor upregulates PPARA and ACSL1 expression in Huh7 cells, while a FOXO1 inhibitor blocks this effect. (O) The AKT inhibitor increases FOXO1 nuclear localisation (*n* = 3).

To further investigate the mechanisms underlying lipid metabolic reprogramming, we conducted a series of in vivo and in vitro experiments. Initially, we measured the levels of FFAs and triglycerides (TGs) in mouse liver tissues during regeneration. The results showed a significant increase in both FFAs and TGs during the early regenerative phase (Figure 5A,B). Subsequent KEGG enrichment analysis of genes in Group 1 (from Figure 4A) revealed significant enrichment in lipid metabolism, peroxisome and PPAR signalling pathways (Figure 5C). Analysis of snRNA‐seq data indicated marked downregulation of Ppara, Ppard and Pparg in proliferating hepatocyte subpopulations (Figure 5D). The Acsl family, comprising Acsl1, Acsl3, Acsl4, Acsl5 and Acsl6, encodes key enzymes catalysing the initial step of fatty acid metabolism. ACSL1 (Acsl1), the most abundant isoform in the liver, activates long‐chain fatty acids by converting them into acyl‐CoA thioesters, thereby channelling substrates towards mitochondrial β‐oxidation, TG synthesis or phospholipid remodelling and plays a central role in maintaining hepatic lipid homeostasis. During early LR, Acsl1 expression was significantly decreased (Figure 5E). snRNA‐seq data further confirmed reduced Acsl1 expression in proliferating hepatocyte clusters (Clusters 3 and 7) (Figure 5F). Western blot analysis also demonstrated a pronounced decrease in Acsl1 protein levels during regeneration (Figures 5G and S7B). GEPIA online website analysis showed that there was a positive correlation between ACSL1 and PPARA in human liver cancer tissues (Figure S6). Bulk transcriptome analysis revealed that, among common Egfr ligands, Areg exhibited the most dramatic change in expression during LR (Figure 5H). IHC staining results showed that AREG expression was elevated after 70% PHx and was primarily expressed in vascular endothelial cells surrounding the PV (portal vein) region. In spatial structure, AREG expression exhibited a high degree of consistency with lipid deposition during the regeneration process (Figure 5I,J). Previous findings from our group indicated that AREG effectively promotes proliferation in the human HCC cell line HuH7. Here, treatment of HuH7 cells with AREG (400 ng/mL) rapidly induced phosphorylation of EGFR and AKT (Figures 5K and S7C). q‐RT‐PCR results showed that AREG significantly suppressed the expression of PPARA and ACSL1 (Figure 5L). Given that FOXO1 has been reported to regulate PPARA and its downstream targets and that AKT‐mediated phosphorylation inhibits FOXO1 nuclear accumulation, we hypothesised that the expression of PPARA and ACSL1 might be modulated through the AKT‐FOXO1 axis. Since HuH7 cells, as a HCC line, inherently exhibit a ‘proliferative hepatocyte’ phenotype with suppressed PPARA and ACSL1 expression compared to normal hepatocytes, we used inhibitors of AKT and FOXO1 to validate this regulatory relationship. q‐RT‐PCR results indicated that AKT inhibitor markedly upregulated PPARA and ACSL1, while a FOXO1 inhibitor completely abolished this upregulation (Figure 5M). Western blot analysis corroborated these findings (Figures 5N and S7D). Finally, immunofluorescence (IF) staining confirmed that AKT inhibition promoted FOXO1 accumulation in the nucleus (Figure 5O). In summary, these results demonstrate that AREG promotes hepatocyte proliferation via enhancing EGFR and AKT phosphorylation. Activated AKT phosphorylates FOXO1, thereby inhibiting the expression of PPARA and ACSL1 and suppressing lipid metabolism. These signalling alterations ultimately lead to the coexistence of enhanced proliferation and suppressed lipid metabolism in regenerating hepatocytes.

### 2.6 Wy‐14643 Promotes Hif1α‐Foxm1 Expression to Accelerate LR

**FIGURE 6:**
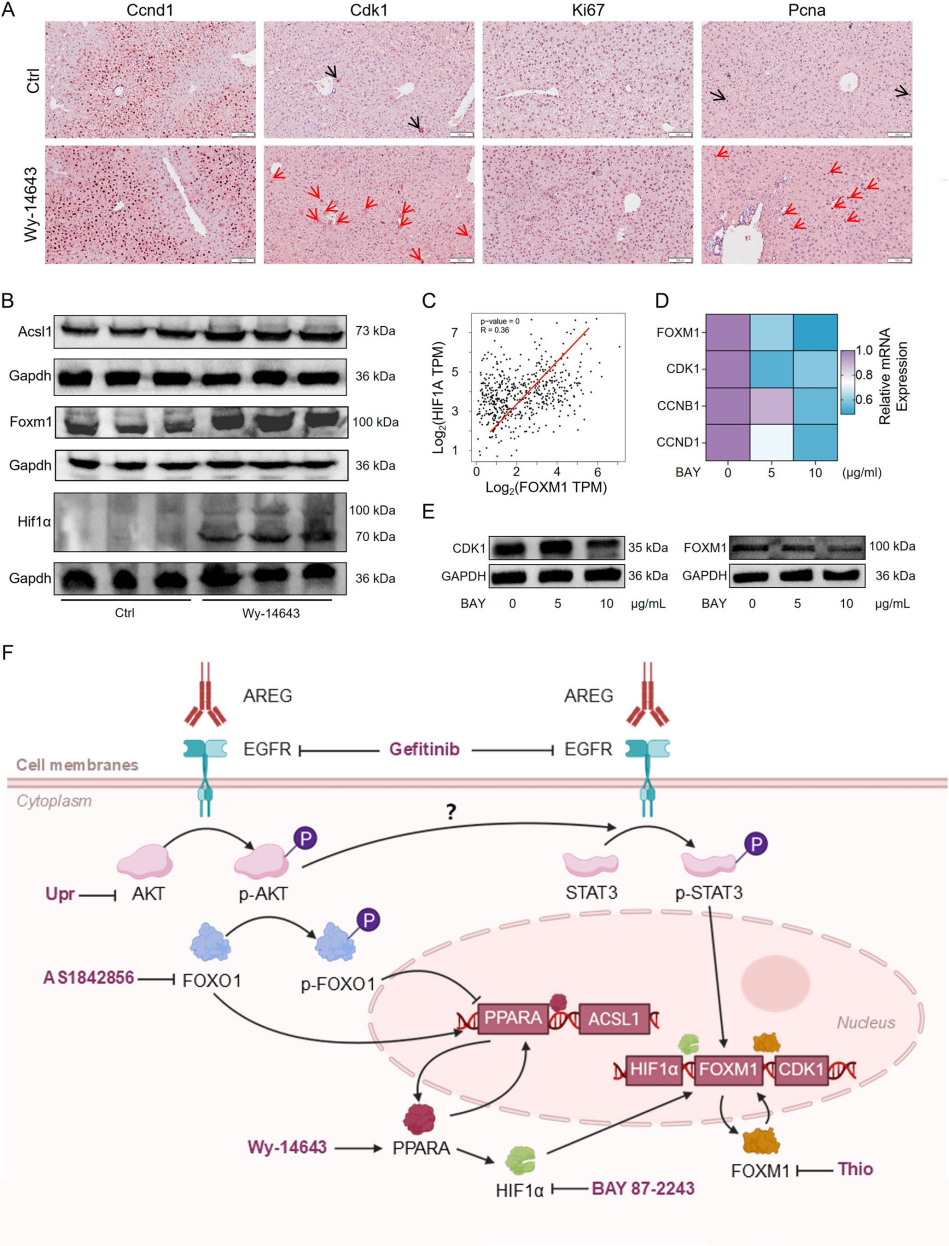
Wy‐14643 promotes hepatocyte proliferation by activating both Ppara‐Acsl1 and Hif1α‐Foxm1 axes. (A) Immunohistochemical results demonstrate that Wy‐14643 effectively enhances LR in mice. (B) Western blot analysis shows that Wy‐14643 upregulates the expression of Acsl1, Foxm1 and Hif1α in regenerating liver tissue after 70% PHx. (C) Analysis using the GEPIA online platform reveals a positive correlation between FOXM1 and HIF1A expression. (D) q‐RT‐PCR results indicate that a Hif1α inhibitor significantly reduces FOXM1 expression. (E) Western blot analysis confirms that HIF1α inhibition effectively suppresses FOXM1 protein expression in HuH7 cell line. (F) Schematic diagram summarising the role of AREG in promoting cell proliferation and suppressing lipid metabolism (*n* = 3).

Although our study has preliminarily clarified the mechanisms underlying hepatocyte proliferation and lipid metabolic reprogramming during LR, how to harness enhanced lipid metabolism to accelerate LR remains a critical question. Double knockout of AKT1 and AKT2 impairs LR, whereas triple knockout including Foxo1 restores regenerative capacity, indicating that functional inhibition of Foxo1 is a crucial initial step for regeneration. Therefore, to therapeutically promote metabolic reprogramming, we targeted downstream effectors of Foxo1—specifically, Ppara and related lipid metabolism genes. Wy‐14643, a well‐characterised and stable agonist of Ppara, was administered via intraperitoneal injection to mice following 70% PHx to investigate its potential to enhance LR and explore the underlying mechanism. Results demonstrated that Wy‐14643 significantly upregulated the expression of CyclinD1, Pcna and Cdk1 in regenerating liver tissues (Figure 6A). Further mechanistic inquiry revealed that Wy‐14643 also promoted the expression of Acsl1, Hif1α and Foxm1, as confirmed by Western blotting (Figures 6B and S7E). Given that both Hif1α and Foxm1 are key TFs regulating LR, we investigated their regulatory relationship. Analysis of HCC data via the GEPIA database showed a strong positive correlation between HIF1A and FOXM1 mRNA expression (Figure 6C). Subsequent in vitro experiments using HuH7 cells treated with the Hif1α inhibitor BAY 87‐2243 demonstrated that Hif1α suppression significantly reduced both FOXM1 and its downstream target CDK1 at both mRNA and protein levels (Figures 6D,E and S7F). In summary, these findings indicate that targeted inhibition of Foxo1‐mediated suppression of lipid metabolism can accelerate LR. Pharmacological activation of Ppara with Wy‐14643 represents a promising therapeutic strategy to enhance LR, operating through a mechanism that involves upregulation of Hif1α and Foxm1.

## 3 Discussion

Hepatocyte proliferation is the fundamental cellular event driving LR. Previous studies have elucidated multiple mechanisms regulating this process [15]. At the hepatocyte level, increased expression of cell cycle‐related proteins serves as a hallmark of proliferation, with key TFs such as Foxm1, Yap1 and HIF1α orchestrating the expression of genes essential for cell division. Further investigations have revealed that activation of receptors on the hepatocyte membrane—including Egfr and Met—triggers crucial kinase signalling pathways such as Pi3k‐Akt, Mapk and Jak‐Stat3, which in turn mediate the functions of these TFs [15]. In recent years, research focus has expanded from hepatocyte‐intrinsic mechanisms to the roles of the regenerative microenvironment and systemic contributors. For instance, vascular endothelial cells sense mechanical stimuli through mechanosensors like Piezo1 or Itgb1, promoting the secretion of hepatocyte mitogens such as Areg, Ereg and Hbegf [2, 6, 7]. Hepatic stellate cells and endothelial cells, both major non‐parenchymal components, have been demonstrated to regulate zonation and functional compartmentalization within hepatic lobules [8, 16, 17]. Additionally, stellate cell‐derived Rspo3 not only modulates hepatocyte zonation but also enhances LR via activation of Wnt signalling [17]. Moreover, glutamate metabolically reprograms bone marrow‐derived macrophages, stabilises HIF1α and transcriptionally upregulates Wnt3, thereby promoting Yap1‐dependent hepatocyte proliferation and supporting regenerative outcomes [2]. Vagus also contribute to this process by modulating kupffer cells to increase Il‐6 secretion, further facilitating LR [5]. Through snRNA‐seq of liver tissues collected at 0 and 48 h after 70% PHx in mice, we identified Egfr‐mediated signalling as a critical pathway underpinning molecular events related to hepatocyte proliferation.

The transcriptional regulation of proliferating hepatocytes during LR is highly complex. Utilising SCENIC analysis to examine regulon activity across distinct hepatocyte subpopulations, we observed that cluster 0 exhibited high activity of Ppara and Pparg regulons. In Cluster 1, Cebpa and Srebf1 were notably active, while cluster 2 was characterised by elevated activity of Zbtb2, Ets1 and Myb. Clusters 3 and 7 displayed heightened regulon activities involving Foxm1, Rad21, E2f7, Mxd3 and Mybl1. These findings indicate that proliferating hepatocytes exhibit enhanced activity of TSs associated with cell proliferation—such as Foxm1, Rad21, E2f7 and Mybl1—while showing relatively reduced activity of metabolism‐related regulators, including Ppara, Pparg, Cebpa and Srebf1. Subsequent analysis of highly expressed genes in Clusters 3 and 7 identified several genes critically involved in cell proliferation and division, such as Bub1b, Bub1, Cdk1, Knl1, Ndc80, Sgo1, Mad2l1, Chek2, Pds5a and Pds5b. Through the analysis of a TF prediction website, results suggested that these genes are potentially regulated by Foxm1. This is further supported by a significant correlation between FOXM1 and these target genes in HCC. In vivo experiments confirmed that pharmacological inhibition of Foxm1 effectively impairs LR and downregulates the expression of key genes including Cdk1.

As evidenced by the aforementioned analysis, proliferating hepatocytes not only undergo proliferation‐associated molecular events but also exhibit suppression of metabolic processes mediated by Ppara, Pparg, Cebpa and Srebf1—key regulators of lipid metabolism [18, 19, 20, 21]. Transcriptomic profiling of regenerating mouse liver tissue revealed extensive metabolic reprogramming, characterised by significant downregulation of pathways related to lipid metabolism, Ppar signalling and peroxisomal function. Consistent with this, scMetabolism analysis based on snRNA‐seq further demonstrated marked inhibition of peroxisomal lipid metabolic processes and fatty acid metabolism within proliferating hepatocytes. During LR, the increase in lipid accumulation is referred to as TRAS. Previous studies have indicated that lipid deposition plays a critical regulatory role in LR. Some research suggests that insufficient lipid accumulation can impair regeneration, while other evidence demonstrates that excessive lipid deposition is also closely associated with regenerative dysfunction [10]. The prevailing view suggests that the uptake of peripheral fatty acids into hepatocytes is a major contributor to lipid accumulation [11, 22]. However, studies employing hepatocyte‐specific Fabp1 knockout mice revealed that while the deletion of Fabp1 significantly reduced LD deposition during LR, it did not markedly affect the overall regenerative process [23]. The relationship between lipid deposition and LR remains highly heterogeneous. Hepatic TG or FFA accumulation can arise from at least five distinct routes: (1) delivery of excess dietary TG via chylomicrons, (2) augmented de novo lipogenesis, (3) overflow of FFA released from adipose tissue lipolysis, (4) impaired very‐low‐density lipoprotein (VLDL)‐mediated export and (5) diminished FFA oxidation. As we summarised previously, these alterations produce three divergent LR phenotypes‐enhanced regeneration, suppressed regeneration or no detectable effect. The underlying scenarios appear to be as follows: (1) impaired LR coincides with increased LD storage. Blocked FFA oxidation (e.g., PPARα loss) simultaneously increases LD content and deprives regenerating hepatocytes of energy, thereby blunting LR. (2) Reduced LD deposition suppresses LR. When hepatic FFA uptake is severely compromised against a backdrop of normal oxidation, insufficient LDs are available for subsequent ATP generation, thereby hampering LR (as illustrated by caveolin‐1 [CAV1] deficiency). (3) Decreased LD storage again inhibits LR, but via export rather than uptake defects. Perilipin‐2 (Plin2) deletion, for instance, accelerates lipid egress, lowering intracellular LD reserves and attenuating regeneration. (4) LD content appears irrelevant to LR. Redundant transporters compensate for loss of a single lipid‐transfer protein and residual LDs meet the modest energetic demands of regeneration. (5) Temporal—rather than absolute—changes in steatosis determine the outcome. Peak TG levels may be identical, yet prolonged retention alters the kinetics; hepatocyte hypertrophy can substitute for proliferation to restore liver mass, as seen in phosphatase and tensin homologue (PTEN)‐deficient mice with heightened oxidative flux. Thus, the interplay between lipid trafficking and storage pathways and LR is intricate; genetically engineered mice often remodel multiple related genes, further confounding any simple link between steatosis and regenerative capacity [10]. Metabolic reprogramming influences LR not only by supplying energy but also through modulating the expression of histones and non‐histone proteins. LD accumulation facilitates the degradation of Mier1 protein, reducing its abundance and thereby promoting the regenerative process [22]. Furthermore, after 70% PHx, a substantial increase in acetyl‐CoA levels enhances histone acetylation, which also serves as a key mechanism driving LR [12]. Although the Ppar signalling pathway is significantly suppressed in proliferating hepatocytes during LR, previous studies have demonstrated that Ppara knockout inhibits the regenerative process, whereas Ppara agonists effectively accelerate it [3, 24]. These findings collectively indicate that Ppara‐mediated gene expression promotes LR.

The underlying mechanism for the suppression of Ppar signalling in proliferating hepatocytes during LR remains unclear. Our analytical data indicate that hepatocytes simultaneously undergo Egfr‐mediated proliferation and Ppar‐mediated suppression of lipid metabolism, suggesting a potential crosstalk between these two pathways. We therefore examined expression changes of Egfr ligands during LR and found that Areg showed the most pronounced alteration. Our previous studies have demonstrated that AREG markedly promotes proliferation in the HCC cell line HuH7 primarily via the EGFR–ERK1/2 signalling pathway [25]. Hence, we further investigated whether AREG could suppress the expression of lipid metabolism‐related genes in HuH7 cells. Indeed, EGFR‐mediated signal transduction represents one of the critical mechanisms underlying the development of transient LR‐associated steatosis. Although the conventional view holds that the transport of peripheral fatty acids constitutes the predominant cause of hepatocellular lipid accumulation during regeneration, some evidence has demonstrated that EGFR inhibition by Canertinib (EGFR and ERBB2) completely abolishes LD formation following 70% PHx [26]. Mechanistic investigations further revealed that EGFR suppression attenuates LD deposition through the inhibition of lipid synthesis. Our study substantiates EGFR involvement in lipid accumulation from the perspective of lipid metabolic regulation, thereby providing complementary theoretical foundations for understanding the pathogenesis of hepatic steatosis during LR.

Our results indicated that after inducing phosphorylation of EGFR and AKT, AREG significantly suppressed the expression of PPARA and ACSL1. Previous studies have shown that both AKT and FOXO1 are critical regulators of hepatocyte lipid metabolism [27]. Phosphorylated AKT promotes phosphorylation of FOXO1 at Thr24 and Ser256, leading to its nuclear export [28]. Inhibition of AKT effectively upregulated the expression of PPARA and ACSL1, an effect that was completely reversed by FOXO1 inhibition. Concurrently, AKT inhibition promoted the accumulation of FOXO1 in the nucleus. These results demonstrate that p‐AKT and FOXO1 are key mediators suppressing the expression of PPARA and its downstream lipid metabolic genes. However, targeting Akt and Foxo1 is not a potential strategy for modulating LR. Studies have shown that dual knockout of Akt1 and Akt2 markedly inhibits LR after 70% PHx, while additional knockout of Foxo1 alleviates this regeneration impairment [29]. In HCC, Foxo1 is recognised as an inhibitor of cancer cell proliferation [29]. Thus, from the perspective of proliferation regulation, the Areg–Egfr–Akt axis exerts a positive regulatory role by suppressing Foxo1 nuclear accumulation. Nevertheless, since Foxo1 is also a key TF for lipid metabolism genes such as Ppara and its downstream targets, its suppression in proliferating hepatocytes effectively becomes a ‘collateral effect or side effect’ of Areg–Egfr‐mediated proliferation [30]. Interestingly, the presence and activation of AKT in the liver appear to function primarily by restraining FOXO1 activity; indeed, deletion of FOXO1 alone fully reverses the pathological sequelae triggered by defective AKT expression [29, 31]. Viral hepatitis, steatohepatitis and related disorders are major drivers of hepatic fibrosis and eventual cirrhosis and fibrosis not only compromises the liver's regenerative capacity but also constitutes the leading risk factor for post‐resection liver failure [32, 33]. AREG secreted by regulatory T cells accelerates fibrogenesis [34], whereas activation of EGFR and AKT likewise propels the fibrotic process; consequently, inhibition of EGFR‐AKT signalling has become a validated strategy for mitigating liver fibrosis [35, 36, 37, 38]. In parallel, PPARα activation has been demonstrated to ameliorate fibrosis [39]. Collectively, these findings reinforce our central conclusion that the AKT‐FOXO1‐PPARα axis represents a promising therapeutic target for enhancing LR–especially in the context of pre‐existing fibrosis. Nevertheless, it must be emphasised that the etiologies underlying hepatic fibrosis are highly heterogeneous and the precise regulatory logic, upstream‐downstream relationships and translational potential of the AKT‐FOXO1‐PPAR axis within this complex milieu remain to be systematically dissected. Such in‐depth studies are essential to provide a solid theoretical foundation and to chart safe, evidence‐based avenues for promoting LR in the clinic.

Given that suppression of lipid metabolism is not a prerequisite for cell proliferation, early targeting of lipid metabolism represents a viable strategy for modulating LR. Combined with previous findings, we propose that the actual intervention targets should lie downstream of Foxo1, such as Ppara and its downstream genes, which also explains why targeting Ppara promotes LR. Previous research has shown that the Ppara agonist Wy‐14643 can regulate cell proliferation via Yap1; our results further demonstrate that Wy‐14643 effectively activates Pparα, induces Acsl1 expression, accelerates lipid metabolism in hepatocytes and upregulates the expression of Hif1α and Foxm1 in the regenerating liver—both of which are important TFs in LR. Further experiments showed that inhibition of Hif1α in HuH7 cells significantly reduced the expression of FOXM1 and CDK1, suggesting that Wy‐14643 may accelerate LR by promoting Hif1α accumulation, which in turn upregulates Foxm1. PPARα is a central transcriptional regulator of lipid metabolism whose downstream target genes are involved in fatty acid transport and oxidation. These oxidation‐related proteins are distributed in mitochondria and peroxisomes; the functional diversity and subcellular localisation of PPARα targets determine the complexity of its cellular effects. On the one hand, PPARα‐mediated transcription of FABP1 alters hepatocytic fatty acid uptake, while on the other, it regulates CPT1A and ACSL1, thereby influencing mitochondrial oxidative phosphorylation [27, 40]. PPARα also modulates peroxisomal fatty acid–metabolising enzymes (ACOX1), thereby perturbing cellular metabolism [27]. In HCC, PPARα downregulation causes ether‐lipid accumulation that enhances cell motility and tumour progression [41]. Low glucose increases AMPK‐dependent phosphorylation of transferrin at Ser685, strengthens its interaction with PPARα, stabilises the protein, boosts fatty acid oxidation and thus promotes HCC progression [42]. Activation of PPARα by hepatitis C virus (HCV) core protein is likewise implicated in virus‐induced hepatocarcinogenesis via elevated fatty acid transport and oxidation [43]. While PPARα fuels lipid metabolism and HCC proliferation, it could also serve as a beneficial target for LR; however, because it governs an extensive set of genes, more stable and precise downstream targets remain to be defined. Although PPARα agonists hold promise as a strategy to enhance LR, the role of PPARα is highly complex and its functions in LR and tumour therapy still require careful consideration and balanced evaluation.

Recent studies on PANoptosis have demonstrated that metabolic stress can directly modulate the balance between cell death and proliferation. PANoptosis is a form of highly inflammatory programmed cell death in which apoptosis, necroptosis and pyroptosis are concurrently activated [44]. It cannot be abolished by blocking any single pathway; instead, it is orchestrated by a multiprotein platform termed the PANoptosome. Accumulating evidence indicates that AREG exerts dual—and seemingly paradoxical—effects on cell survival: it can either drive or restrain cell growth depending on cellular context and microenvironmental cues [25, 45, 46]. Our observations that AREG enhances proliferation while concurrently suppressing metabolism may reflect this bifurcation. On the one hand, EGFR activation downstream of AREG upregulates FOXM1 to accelerate proliferation; on the other, the resulting metabolic brake can impose bioenergetic stress that primes the PANoptosome and tips cells towards PANoptosis. The final phenotypic outcome is thus dictated by the balance between these two arms and by the cell's adaptive capacity to rewire its metabolic network. These mechanisms warrant further in‐depth investigation.

In summary, this study further investigates the link between cell proliferation and metabolic reprogramming in proliferating hepatocytes. Our results indicate that activation of Egfr is crucial for quiescent hepatocytes to re‐enter the cell cycle, undergo proliferation and acquire stem‐like properties. Upon EGFR activation, key TFs such as Foxm1 are upregulated, promoting the expression of genes essential for cell proliferation. Concurrently, Egfr signalling enhances phosphorylation of the Akt–Foxo1 axis, leading to suppression of the Foxo1‐mediated Ppar–Acsl1 pathway and resulting in significant inhibition of lipid metabolism in proliferating hepatocytes. Administration of the Ppara agonist Wy‐14643 effectively restored Acsl1 expression in the regenerating liver, accelerating lipid metabolism. Furthermore, PPARA activation enhanced the Hif1α–Foxm1 axis, synergizing with the Areg–Egfr pathway to promote LR (Figure 6F). It is important to note a limitation of the present study. Our mechanistic investigations were conducted partly in the Huh7 hepatoma cell line. Although this model provided a valuable platform for the initial dissection of the EGFR–Akt–FOXO1–PPARα pathway, the translational relevance of these findings to normal liver physiology may be constrained by the transformed nature of this cell line. Future studies employing primary quiescent hepatocytes or more physiologically relevant ex vivo models will be essential to validate and extend our conclusions regarding the role of this signalling axis in normal hepatic regeneration and metabolic regulation.

## 4 Materials and Methods

### 4.1 Animal Care and Surgeries

Male C57BL/6 mice (20–25 g, Charles River) were housed at the Experimental Animal Center of the First Hospital of Jilin University under a 12 h light/12 h dark cycle with adlibitum access to standard chow and water; all procedures were approved by the Animal Ethics Committee of the Experimental Animal Center of the First Hospital of Jilin University (JDYY20240408). Briefly, mice were anaesthetised using isoflurane and an incision was made in the anterior midline of the abdominal wall. The left lateral and median hepatic lobes were individually ligated with 4‐0 silk and resected to achieve 70%. For LR kinetic analysis, 28 mice were randomised into seven groups (*n* = 4) of sham‐operated or PHx animals sacrificed at 12, 18, 24, 48, 72 or 120 h post‐surgery. For pharmacological studies, nine or six additional mice were assigned to receive intraperitoneal gefitinib (80 mg/kg, *n* = 3), thiostrepton (17 mg/kg, *n* = 3), pirinixic acid (Wy‐14643, 50 mg/kg, *n* = 3) or corresponding vehicle every 12 h beginning 24 h before PHx and continuing until 48 h (gefitinib/thiostrepton) or 24 h (Wy‐14643) after surgery, whereupon regenerating liver tissue was harvested. To assess AREG expression and lipid deposition in liver tissue spatially, female mice were subjected to 70% PHx and tissue samples were collected at 48 h post‐surgery for AREG expression analysis (Our previous research has demonstrated that female mice exhibit more pronounced lipid deposition following surgery).

### 4.2 Materials

Uprosertib (GSK2141795) (Upr) (CAS No.: 1047634‐65‐0), Gefitinib (Gef) (CAS No.: 184475‐35‐2), Thiostrepton (Thio) (CAS No.: 1393‐48‐2), Pirinixic acid (Wy‐14643) (CAS No.: 50892‐23‐4), AS1842856 (AS) (CAS No.: 836620‐48‐5), BAY 87‐2243 (BAY) (CAS No.: 1227158‐85‐1) were purchased from MedChemExpress (USA). Recombinant Amphiregulin (AREG) (262‐AR‐100) was acquired from R & D Systems.

### 4.3 Cell Line and Cell Culture

The human hepatoma cell line, HuH7, was cultured in DMEM with 10% Dulbecco's modified Eagle's medium supplemented with 10% foetal bovine serum and 1% penicillin–streptomycin at 37°C in a 5% CO2 incubator. The cells underwent subculturing at intervals of every two to three days.

### 4.4 IHC and IF Staining Procedures

Liver specimens were fixed in 4% paraformaldehyde for 24 h, paraffin‐embedded and serially sectioned at 4 μm. After deparaffinisation and rehydration, antigen retrieval was performed in citrate buffer (pH 6.0) or Tris‐EDTA buffer (pH 9.0 for AREG) at 95°C–100°C for 15 min. Endogenous peroxidase was quenched with 3% H_2_O_2_ for 10 min and non‐specific binding blocked with 10% normal house serum for 60 min at room temperature. The staining process was conducted using a Vector kit (Vector Laboratories) as per the instructions provided by the manufacturer. Primary antibodies comprised rabbit anti‐CDK1 (1:1000; Abcam, ab32094), mouse anti‐PCNA (1:1000; Abcam, ab29), rabbit anti‐Ki67 (1:1000; Abcam, ab15580), rabbit anti‐Cyclin D1 (1:1000; Abcam, ab16663), rabbit anti‐AREG (1:500, Proteintech, 16036‐1‐AP).

For cell IF, about 6000 cells per well were plated into 96‐well plates and allowed to attach overnight. After experimental treatment, the cells were fixed with 4% paraformaldehyde for 15 min at room temperature and then permeabilised with 0.3% Triton in TBST for 10 min. Non‐specific binding was blocked with 5% goat serum for 1 h at room temperature. The cells were then incubated with FOXO1 (Proteintech: 18592‐1‐AP, 1:100 diluted in 5% goat serum and 0.3% Triton X‐100 in TBST) overnight at 4°C. After washing with PBS three times for 5 min each, the cells were incubated with secondary antibody at room temperature for 1 h. Nuclei were stained with DAPI for 15 min and then the cells were washed with PBS three times for 5 min each. The fluorescence microscope was used to photograph and image the cells after adding 50 μL of PBS per well.

### 4.5 Western Blot

Proteins were extracted from cell lines and liver tissue using radioimmunoprecipitation assay buffer with a protease and phosphatase inhibitor cocktail (Beyotime, China). The proteins were separated by 10% SDS‐PAGE gel and transferred to a PVDF membrane using wet transfer method at 120 V for 1.5 h. The membrane was then blocked with 5% fat‐free milk at room temperature for 1 h. Primary antibodies were added and incubated at 4°C overnight. After washing the membrane with TBST buffer three times for 5 min each time, an HRP‐conjugated secondary antibody was added at an appropriate dilution of about 1:1000 for 2 h at room temperature. Bands were detected using enhanced chemiluminescence reagent (Applygen, China). GAPDH and β‐Actin were used as internal references. The antibodies used for western blot testing included: anti‐FOXM1 (Abcam #ab180710, 1:1000), anti‐p‐EGFR (ABclonal #AP0820, 1:1000), anti‐EGFR (Servicebio #GB111504‐100, 1:1000), anti‐ACSL1 (Proteintech #13989‐1‐AP, 1:1000), anti‐p‐AKT (Proteintech # 66444‐1‐Ig, 1:1000), anti‐HIF1α (Abcam ab179483, 1:1000), anti‐CDK1 (Abcam #ab32094, 1:1000), anti‐PPARA (Boster A00600‐2, 1:1000), anti‐GAPDH (Servicebio GB15004, 1:1000) and anti‐β‐Actin (Servicebio GB11003, 1:1000).

### 4.6 Single Cell Nuclear Transcriptome Sequencing

The right lobe liver tissues of mice at 0 and 48 h after 70% PHx were used for single cell nuclear transcriptome sequencing. After tissue acquisition, the liver tissue was washed with pre‐cooled PBS to remove the blood and other tissues on the surface and then placed in a freezer tube for liquid nitrogen quick freezing and then transferred to a −80°C refrigerator for storage. Single cell nuclear transcriptome sequencing was performed by Beijing SeekGene BioSciences Co. Ltd. (Beijing, China). Single cell nuclear transcriptome data analysis was performed on the cloud platform of Beijing SeekGene BioSciences Co. Ltd. (https://seeksoul.online/index.html#/sgvps/home). Previously reported cell‐type marker genes were employed for cluster annotation and UMAP clustering was performed at a resolution of 0.2. The scMetabolism was used to analyse the changes of single‐cell metabolism‐related genes and the Reactome gene set was used as a reference for metabolic gene mapping. Gene Set Variation Analysis (GSVA) was used to analyse different signalling pathways in different hepatocyte populations. SCENIC was used to analyse the differences in TF activity among different hepatocyte populations. Monocle2 was used to perform pseudo‐time series analysis on different groups of hepatocytes. CytoTRACE was used to analyse the differences in the characteristics of hepatocyte stemness in different groups.

### 4.7 Bulk RNA Sequencing Assay

Publicly available RNA‐sequencing data were downloaded from the NCBI Gene Expression Omnibus (GEO) under accession number GSE215423. Differentially expressed mRNAs (DEGs) were defined by |log_2_ fold‐change| ≥ 1.0 and *p* ≤ 0.05. Gene Ontology (GO) and Kyoto Encyclopaedia of Genes and Genomes (KEGG) pathway enrichment analyses were performed with DAVID (https://davidbioinformatics.nih.gov). Putative TFs targeting key DEGs were predicted using the TFpredict web server (https://jingege.shinyapps.io/TF_predict). All visualisations were generated with BioLadder (https://www.bioladder.cn) to ensure accurate and publication‐quality graphics.

### 4.8 Quantitative Reverse Transcription Polymerase Chain Reaction (q‐RT‐PCR)

Total RNA was isolated from cells with TRIzol reagent (Invitrogen, Carlsbad, CA, USA); cDNA was synthesised with the ReverTra Ace qPCR RT Kit (Toyobo, FSQ‐301, Japan) according to the manufacturer's instructions. qPCR was performed on a Bio‐Rad CFX96 system using the SYBR Green Real‐time PCR Master Mix (Toyobo, QPS‐201, Japan). Relative mRNA abundance was calculated by the 2^−ΔΔ*C*t^ method and normalised to β‐actin. All primers (Ruibiotech, Beijing, China) are listed in Table S1.

### 4.9 Quantitative Detection of FFAs and TGs in Liver Tissue

FFA and TG levels in the regenerating lobe were quantified with the Nanjing Jiancheng Bioengineering Institute kits A042‐2‐1 and A110‐1‐1, respectively. Accurately weighed tissue samples were collected from the regenerating liver lobe at 0, 12, 24, 48 and 72 h after 70% PHx, processed strictly according to the manufacturer's instructions and normalised to total protein concentration.

### 4.10 Statistical Analysis

In this study, all experimental data were presented as means ± standard error (SE) from at least three independent experiments. Statistical analysis was performed using GraphPad Prism 8 software. For comparison of data between two different treatment groups, a two‐tailed, unpaired Student's *t*‐test was performed. Differences were considered to be statistically significant when *p* < 0.05. The graphs display data as means ± standard error of the mean (SEM). Statistical significance is denoted as follows: *p ≤ 0.05, **p ≤ 0.01, ***p ≤ 0.001, ****p ≤ 0.0001, while no statistical significance is indicated by ‘ns.’.

## Author Contributions

Conceptualization: Yuelei Hu, Ruilin Wang, Ni An, Jinmei Diao, Juan Liu and Guoyue Lv. Experiment and design, collection and assembly of data: Yuelei Hu, Ruilin Wang, Ni An and Jinmei Diao. Article writing and revise: Yuelei Hu, Shifei Song, Ruilin Wang and Juan Liu. Review and editing of article: Juan Liu and Guoyue Lv.

## Funding

This work was supported by the National Natural Science Foundation of China (32371477 and 82572451); National Key Research and Development Programme of China (No. 2024YFE0213800); Doctor of excellence programme from The First Hospital of Jilin University (JDYY‐DEP‐2024012).

## Ethics Statement

All experimental protocols were approved by the Animal Ethics Committee of the First Hospital of Jilin University (JDYY20240408).

## Consent

The authors have nothing to report.

## Conflicts of Interest

The authors declare no conflicts of interest.

## Supporting information


**Figure S1:** snRNA‐seq reveals the dynamic cellular landscape and identifies a proliferating hepatocyte subpopulation after 70% PHx in mice.
**Figure S2:** Pseudotime plots showing distribution of each hepatocytes cluster along combined cellular trajectories shown in (A).
**Figure S3:** FeaturePlots depicting regulon activity based on RSS calculation.
**Figure S4:** Transcription factor prediction of cell division related genes.
**Figure S5:** Lipid metabolism inhibition occurred in the early stage of liver regeneration in mice.
**Figure S6:** The relationship between ACSL1 and PPARA, PPARD and PPARG.
**Figure S7:** Relative quantitative analysis of Western Blot results.
**Table S1:** List of primer sequences used for q‐RT‐PCR.

## Data Availability

The data that support the findings of this study are available from the corresponding author upon reasonable request.
