# Supplementary material for: Targeting Egfr‐Mediated Cell Proliferation and Lipid Metabolism Separation Effectively Accelerate Liver Regeneration
Source: Cell Prolif. 2026 Apr 22:e70214. Online ahead of print. doi: 10.1111/cpr.70214 (PMC13325914; doi:10.1111/cpr.70214)
Supplement: Supplementary file 1 — Figure S1: snRNA‐seq reveals the dynamic cellular landscape and identifies a proliferating hepatocyte subpopulation after 70% PHx in mice. Figure S2: Pseudotime plots showing distribution of each hepatocytes cluster along combined cellular trajectories shown in (A). Figure S3: FeaturePlots depicting regulon activity based on RSS calculation. Figure S4: Transcription factor prediction of cell division related genes. Figure S5: Lipid metabolism inhibition occurred in the early stage of liver regeneration in mice. Figure S6: The relationship between ACSL1 and PPARA, PPARD and PPARG. Figure S7: Relative quantitative analysis of Western Blot results. Table S1: List of primer sequences used for q‐RT‐PCR. [file CPR-9999-e70214-s001.docx]

**Supporting Information**


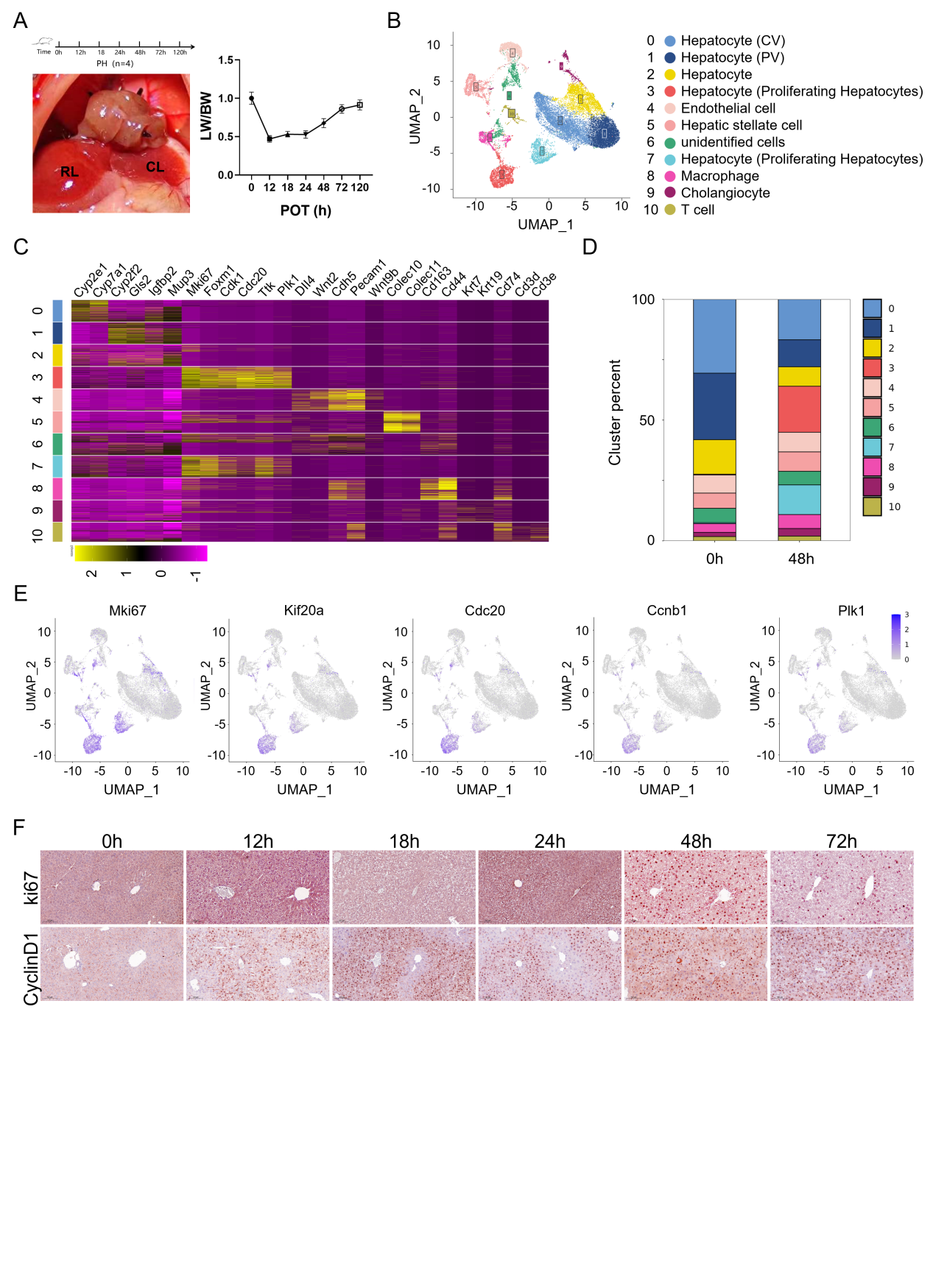


**Supplementary figure 1**. snRNA-seq reveals the dynamic cellular landscape and identifies a proliferating hepatocyte subpopulation after 70% PHx in mice. A. Schematic illustration of the 70% PHx model (n=4). B UMAP projection of snRNA-seq data from liver tissues at 0 and 48 hours post-PHx, showing the identification of major cell types. C. Heatmap displaying the expression of canonical marker genes used to define each cell cluster identified in (B). D. Bar plots quantifying the proportional changes in the abundance of each major cell cluster between the 0h and 48h time points post-PHx. E. Analysis of proliferative gene signatures. F. Immunohistochemical (IHC) validation of hepatocyte proliferation in liver sections post-PHx.


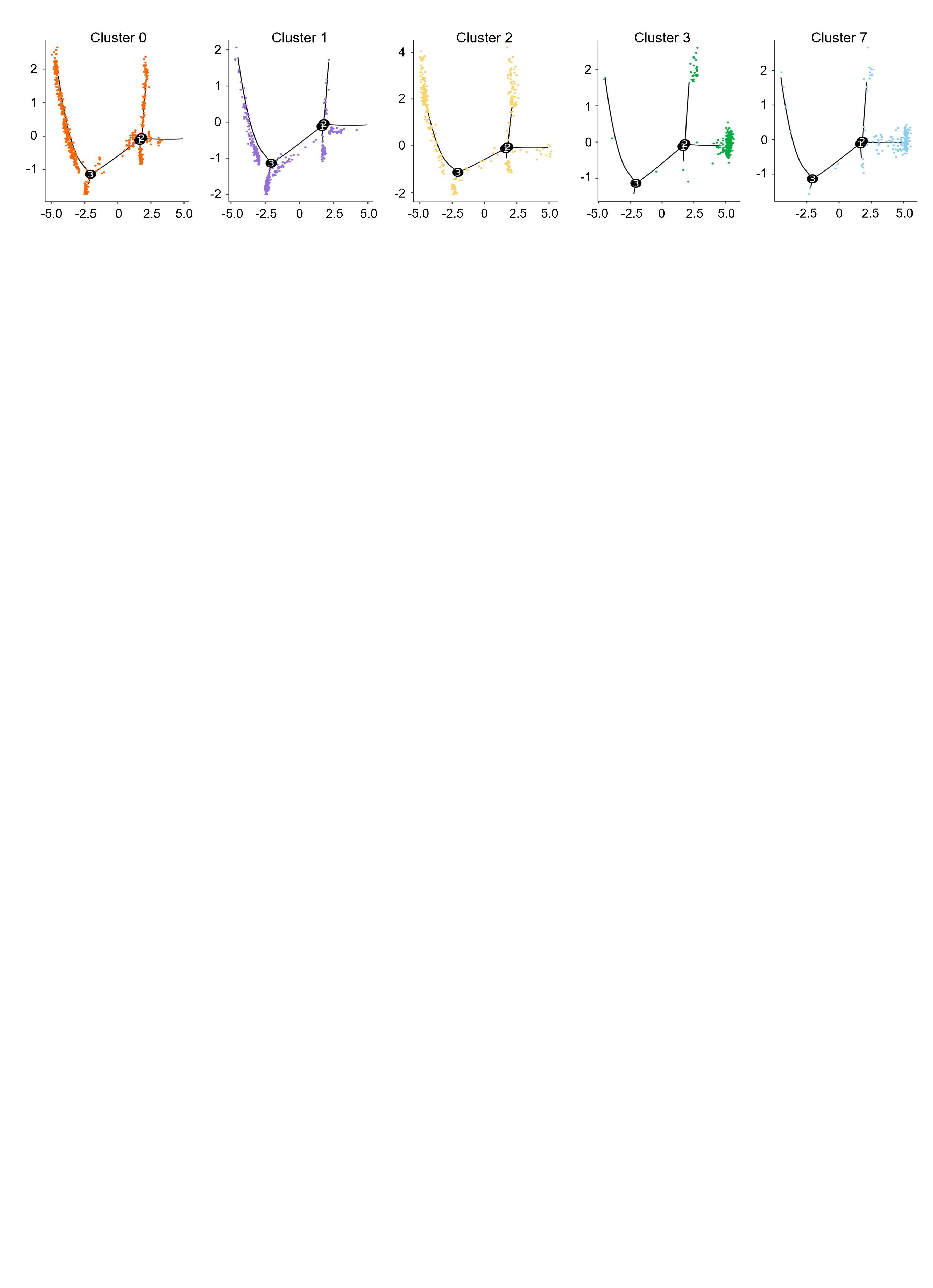


**Supplementary figure 2**. Pseudotime plots showing distribution of each hepatocytes cluster along combined cellular trajectories shown in (A).


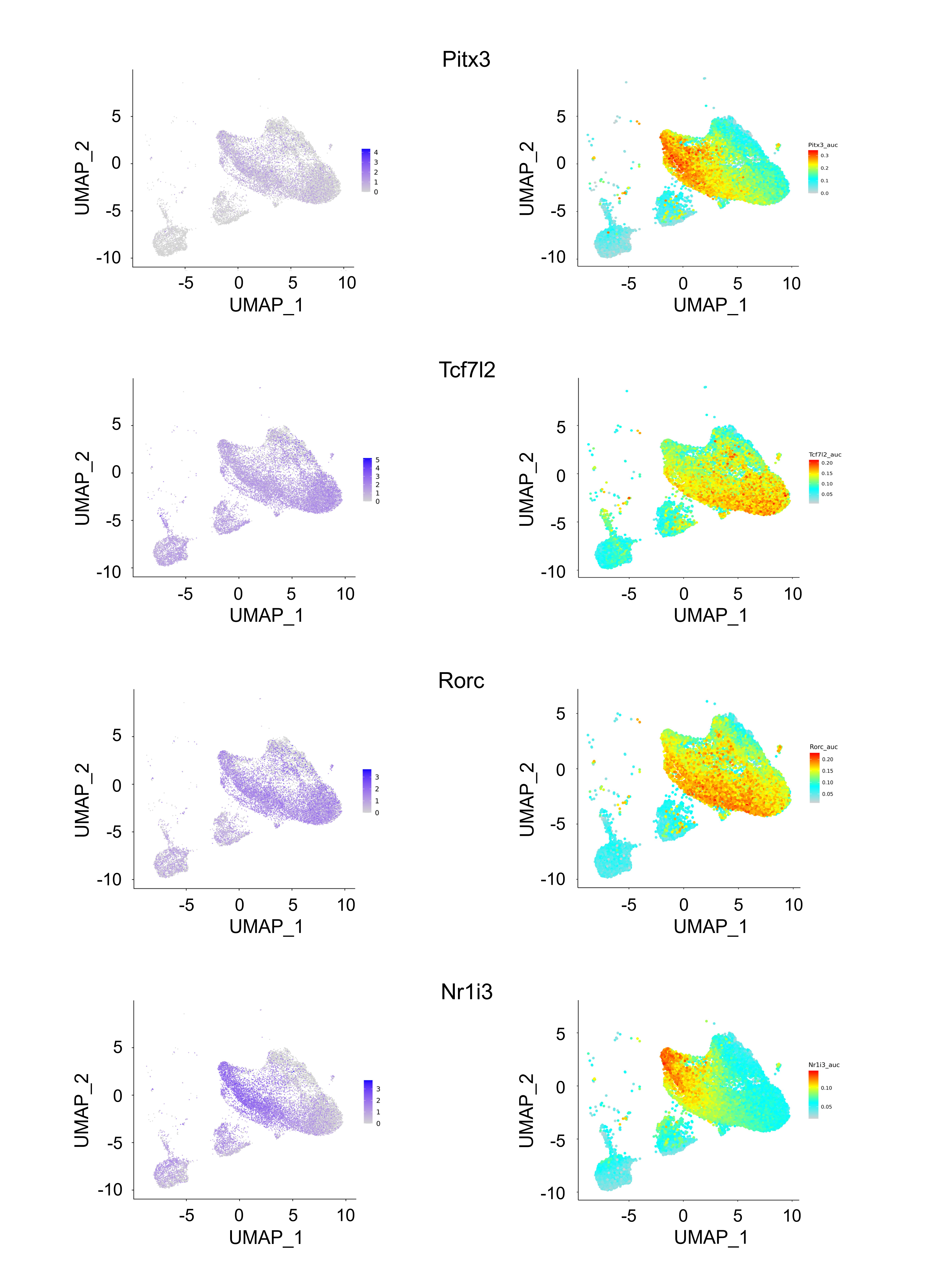


**Supplementary figure 3.** FeaturePlots depicting Regulon activity based on RSS calculation. FeaturePlot visualizing the expression and AUC values of selected Regulons in non-proliferating hepatocytes, calculated based on RSS.


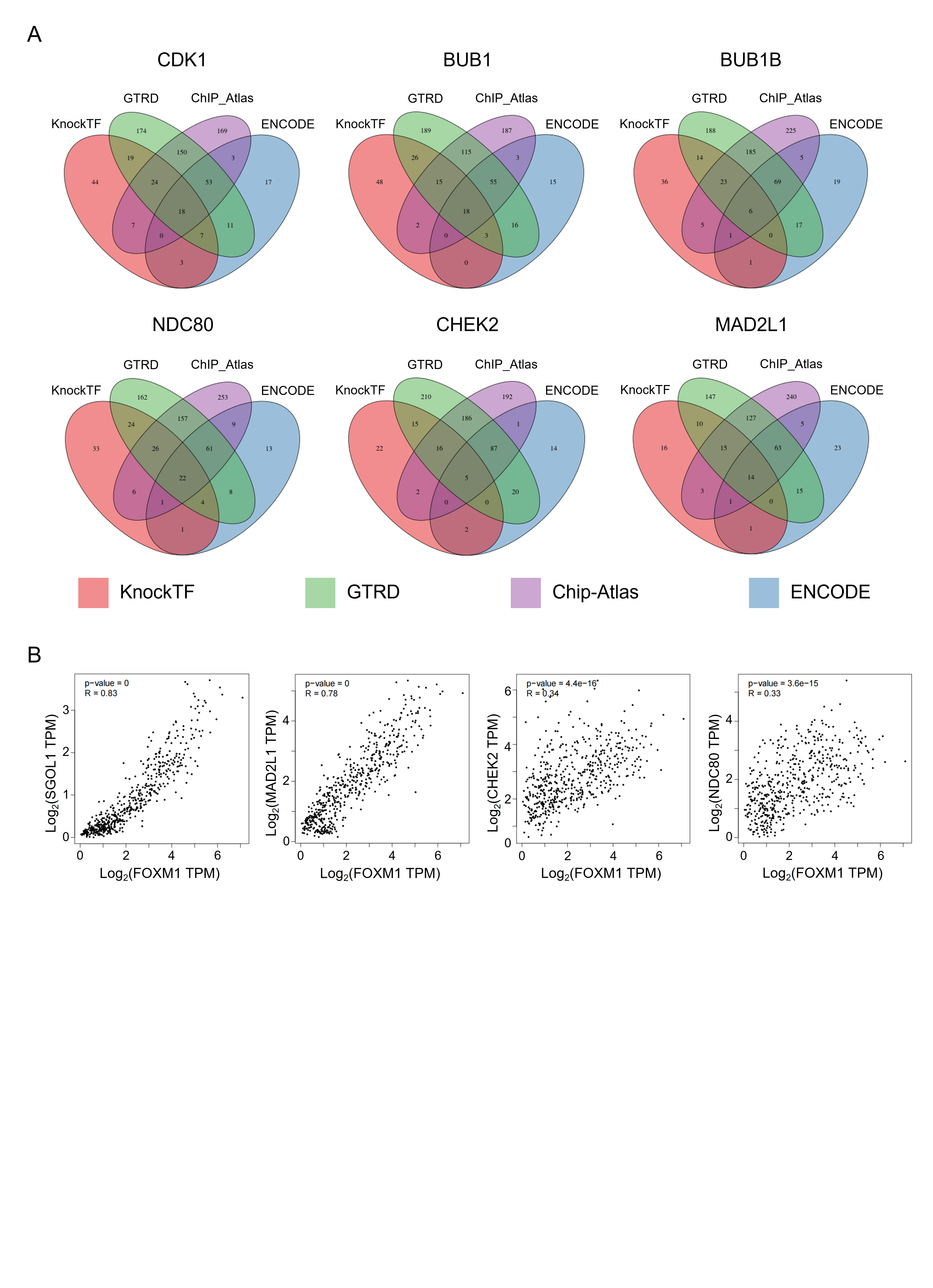


**Supplementary figure 4**. Transcription factor prediction of cell division related genes. A. The online transcription factor prediction website predicts transcription factors of key genes related to cell division based on KnockTF, GTRD, Chip-Atlas and ENCODE databases. B. Correlation analysis between Foxm1 and the key target genes in human HCC tissue samples from the GEPIA database.


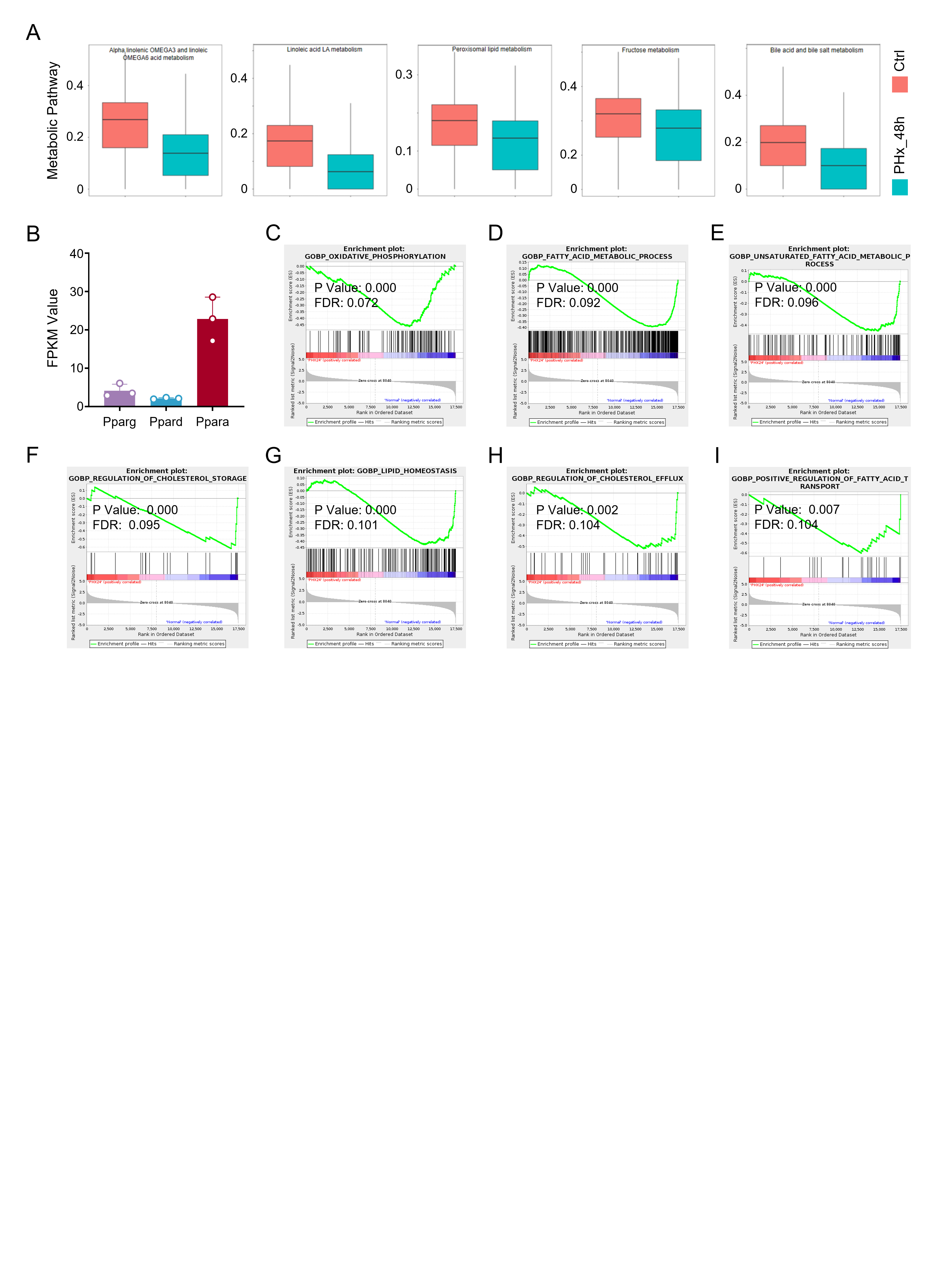


**Supplementary figure 5.** Lipid metabolism inhibition occurred in the early stage of liver regeneration in mice. A. Bulk_RNA seq data revealed the difference in the expression level of Ppara, Ppard and Pparg family in mouse liver tissue. B-H. Gene Set Enrichment Analysis (GSEA) confirms the inhibition of lipid metabolism pathways following 70% PHx, comparing 24h and 0h time points. I. The box plot showed the difference of representative metabolic pathways in single cell nuclear transcriptome sequencing between the two groups at 0h and 24h.


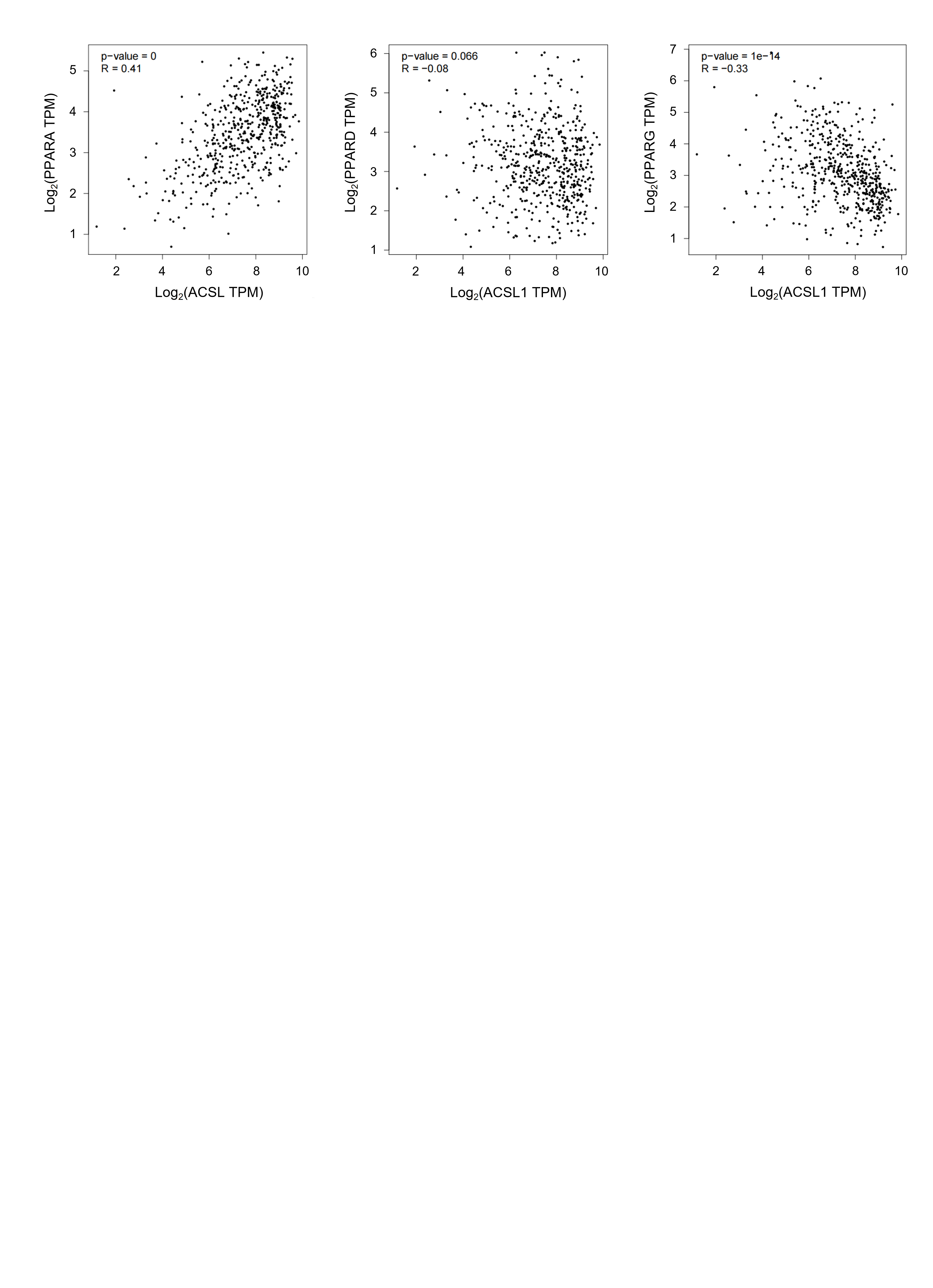


**Supplementary figure 6.** The relationship between ACSL1 and PPARA, PPARD and PPARG. GEPIA online website analysis showed that there was a positive correlation between ACSL1 and PPARA in human liver cancer tissues.


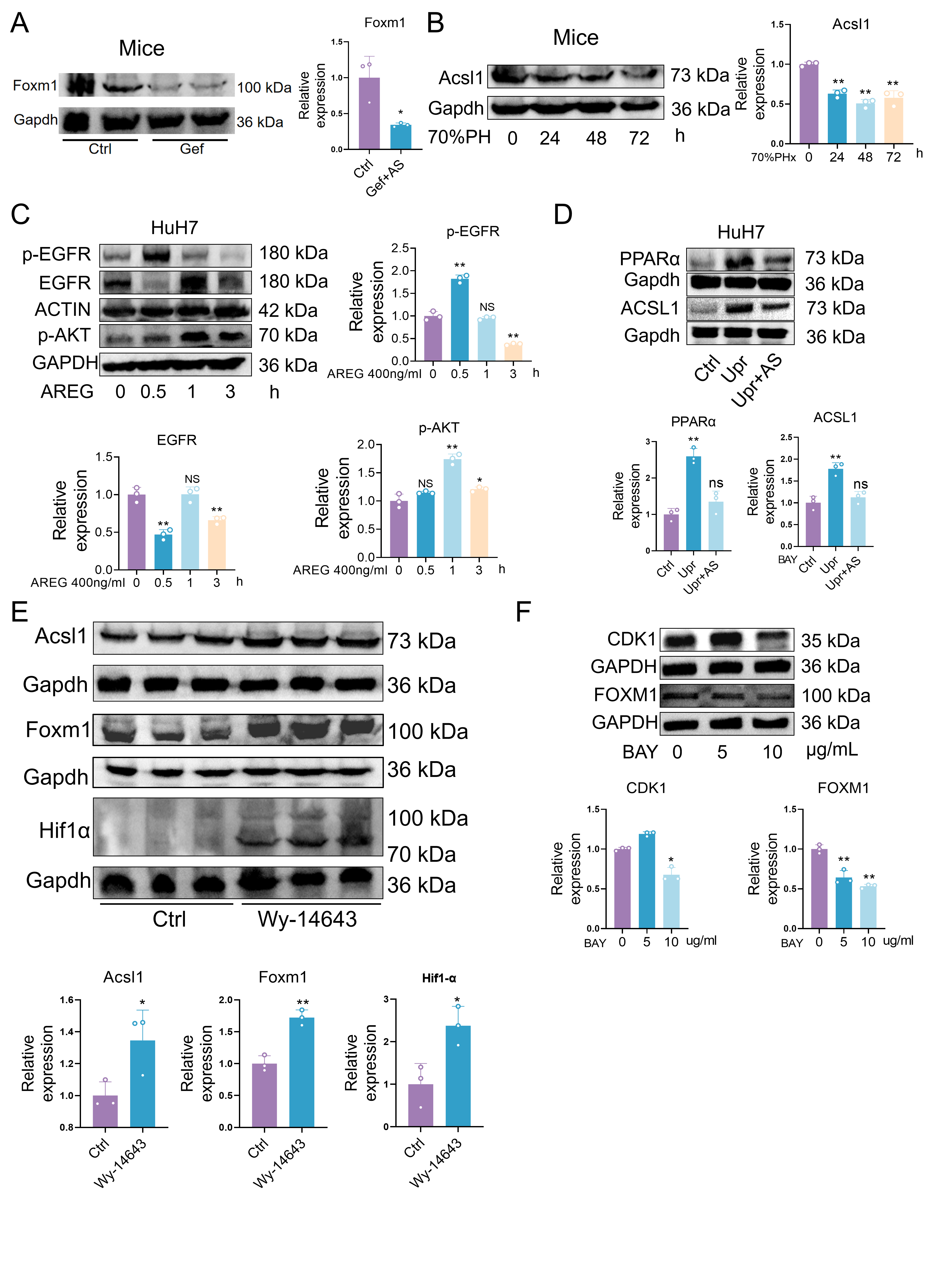


**Supplementary figure 7.** Relative quantitative analysis of Western Blot results. A. Gefitinib inhibits Foxm1 expression during liver regeneration (Figure 3G). B. Acsl1 expression levels are downregulated during liver regeneration (Figure 6G). C. AREG activates EGFR and AKT phosphorylation in HuH7 cells (Figure 6K). D. AKT and FOXO1 are involved in regulating the expression of PPARA and ACSL1 (Figure 6N). E. PPARα agonist Wy-14643 promotes the expression of Acsl1, Foxm1, and Hif1α (Figure 7B). F. Inhibition of Hif1α reduces the expression of CDK1 and FOXM1 (Figure 7E).

**Supplementary table 1.** List of primer sequences used for q-RT-PCR

|  | Forward | Reverse |
| --- | --- | --- |
| FOXM1 | CGTCGGCCACTGATTCTCAAA | GGCAGGGGATCTCTTAGGTTC |
| CDK1 | AAACTACAGGTCAAGTGGTAGCC | TCCTGCATAAGCACATCCTGA |
| PPARA | ATGGTGGACACGGAAAGCC | CGATGGATTGCGAAATCTCTTGG |
| ACSL1 | CGACGAGCCCTTGGTGTATTT | GGTTTCCGAGAGCCTAAACAA |
| CCND1 | GCTGCGAAGTGGAAACCATC | CCTCCTTCTGCACACATTTGAA |
| CCNB1 | AATAAGGCGAAGATCAACATGGC | TTTGTTACCAATGTCCCCAAGAG |
| ACTB | CCTGGCACCCAGCACAAT | GGGCCGGACTCGTCATAC |
